# COVID-19: Proposing a Ketone-Based Metabolic Therapy as a Treatment to Blunt the Cytokine Storm

**DOI:** 10.1155/2020/6401341

**Published:** 2020-09-09

**Authors:** Patrick C. Bradshaw, William A. Seeds, Alexandra C. Miller, Vikrant R. Mahajan, William M. Curtis

**Affiliations:** ^1^Department of Biomedical Sciences, Quillen College of Medicine, East Tennessee State University, Johnson City, TN 37614, USA; ^2^Seeds Scientific Research & Performance, Spire Institute, Geneva, OH 44041, USA; ^3^Science Research Department, Armed Forces Radiobiology Research Institute, Uniformed Services University of the Health Sciences, Bethesda, MD 20889, USA; ^4^Center for Radiological Research, Columbia University Medical Center, New York, NY 10036, USA; ^5^Vanderbilt University, Nashville, TN 37240, USA

## Abstract

Human SARS-CoV-2 infection is characterized by a high mortality rate due to some patients developing a large innate immune response associated with a cytokine storm and acute respiratory distress syndrome (ARDS). This is characterized at the molecular level by decreased energy metabolism, altered redox state, oxidative damage, and cell death. Therapies that increase levels of (R)-beta-hydroxybutyrate (R-BHB), such as the ketogenic diet or consuming exogenous ketones, should restore altered energy metabolism and redox state. R-BHB activates anti-inflammatory GPR109A signaling and inhibits the NLRP3 inflammasome and histone deacetylases, while a ketogenic diet has been shown to protect mice from influenza virus infection through a protective *γδ* T cell response and by increasing electron transport chain gene expression to restore energy metabolism. During a virus-induced cytokine storm, metabolic flexibility is compromised due to increased levels of reactive oxygen species (ROS) and reactive nitrogen species (RNS) that damage, downregulate, or inactivate many enzymes of central metabolism including the pyruvate dehydrogenase complex (PDC). This leads to an energy and redox crisis that decreases B and T cell proliferation and results in increased cytokine production and cell death. It is hypothesized that a moderately high-fat diet together with exogenous ketone supplementation at the first signs of respiratory distress will increase mitochondrial metabolism by bypassing the block at PDC. R-BHB-mediated restoration of nucleotide coenzyme ratios and redox state should decrease ROS and RNS to blunt the innate immune response and the associated cytokine storm, allowing the proliferation of cells responsible for adaptive immunity. Limitations of the proposed therapy include the following: it is unknown if human immune and lung cell functions are enhanced by ketosis, the risk of ketoacidosis must be assessed prior to initiating treatment, and permissive dietary fat and carbohydrate levels for exogenous ketones to boost immune function are not yet established. The third limitation could be addressed by studies with influenza-infected mice. A clinical study is warranted where COVID-19 patients consume a permissive diet combined with ketone ester to raise blood ketone levels to 1 to 2 mM with measured outcomes of symptom severity, length of infection, and case fatality rate.

## 1. Introduction

There are tremendous demands on governments and the private sector to solve the COVID-19 crisis with an effective and timely vaccine or therapy. As time passes, the demand for information grows pertaining to how healthy lifestyle and nutrition may play a role in protection against the detrimental outcomes of the SARS-CoV-2 virus. In this review, the intricate and detailed interplay among nutrition, metabolism, and the tightly controlled immune system is highlighted. The data suggest that exogenous ketones can increase cell efficiency and metabolic flexibility to provide significant immune modulation. However, challenges remain in identifying the exact dietary macronutrient combinations that will best influence the immune system. It is important for researchers and clinicians to consider metabolic strategies when attempting to identify novel preventative measures for viral infection, as these therapies can support the patient's immune system while showing minimal toxicities. The mechanisms through which exogenous ketones improve energy and redox metabolism and blunt inflammation likely apply not only to COVID-19 but to any viral or bacterial infection where excessive cytokine production can lead to multiple organ failure and mortality. There are many types of metabolic therapies. However, therapies that increase R-BHB levels, including the consumption of a ketogenic diet or different forms of exogenous ketones, will be the focus of this review. Others have also suggested that increasing systemic ketone levels may aid host defenses against respiratory viral infection, in part, by decreasing inflammation [1, 2], including a recent comprehensive review [3], while a clinical trial of the effects of a ketogenic diet on intubated SARS-CoV-2 patients has recently been registered (NCT04358835).

### 1.1. SARS-CoV-2 Infects Type II Alveolar Epithelial Cells and Induces the Innate and Acquired Immune Responses

**Figure 1:**
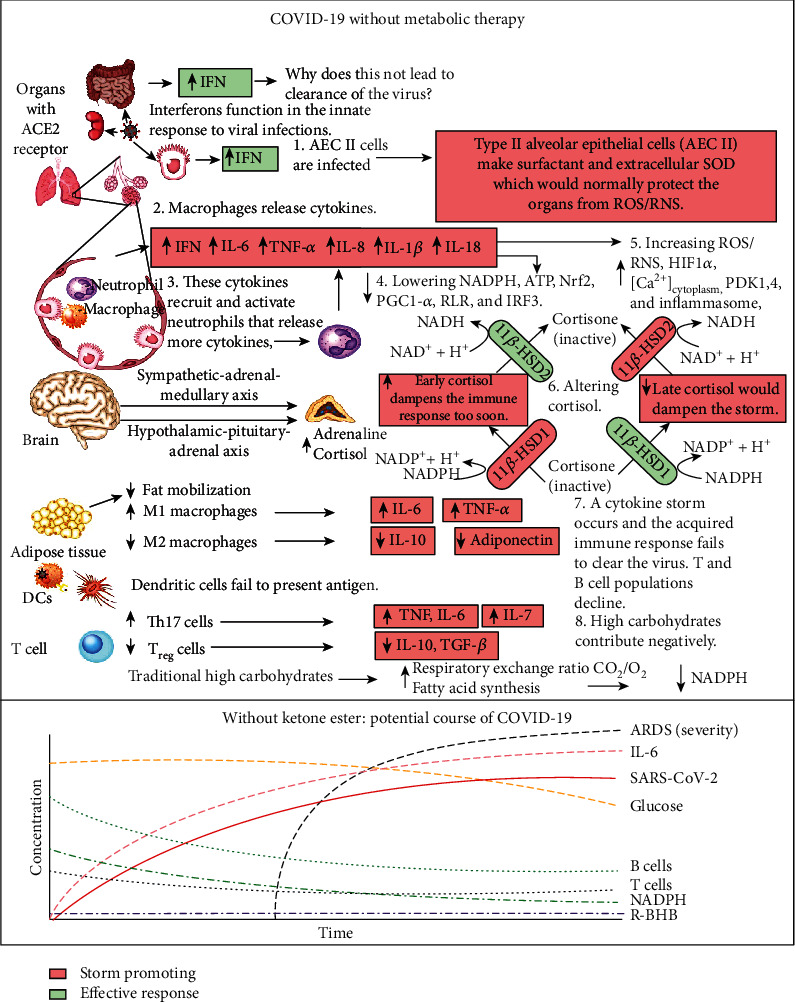
Mechanisms that lead to acute respiratory distress syndrome (ARDS) and mortality following SARS-CoV-2 infection are shown. The cells of the innate immune response secrete increasing amounts of cytokines. The cells that normally protect against a cytokine storm lose this ability leading to a runaway positive feedback loop of cytokine production. Abbreviations: 11*β*-HSD1 and 11*β*-HSD2: 11*β*-hydroxysteroid dehydrogenase types 1 and 2; ACE2: angiotensin-converting enzyme 2; AEC I and AEC II: alveolar epithelial cell types I and II; DCs: dendritic cells; FOXO1: forkhead box O1 transcription factor; FOXO3: forkhead box O3 transcription factor; HIF-1*α*: hypoxia-inducible factor 1 alpha; IFN: interferon; IL-6: interleukin-6; IRF3: IFN-regulatory factor 3; NAD(H): nicotinamide adenine dinucleotide; NADP(H): nicotinamide adenine dinucleotide phosphate; ONOO^−^: peroxynitrite; PGC1-*α*: PPARG coactivator 1-alpha; PDK1 and PDK4: pyruvate dehydrogenase kinases 1 and 4; RLR: retinoic acid-inducible gene I-like receptors; RNS: reactive nitrogen species; ROS: reactive oxygen species; SOD: superoxide dismutase; TGF-*β*: transforming growth factor-*β*; TNF-*α*: tumor necrosis factor-alpha.

**Figure 2:**
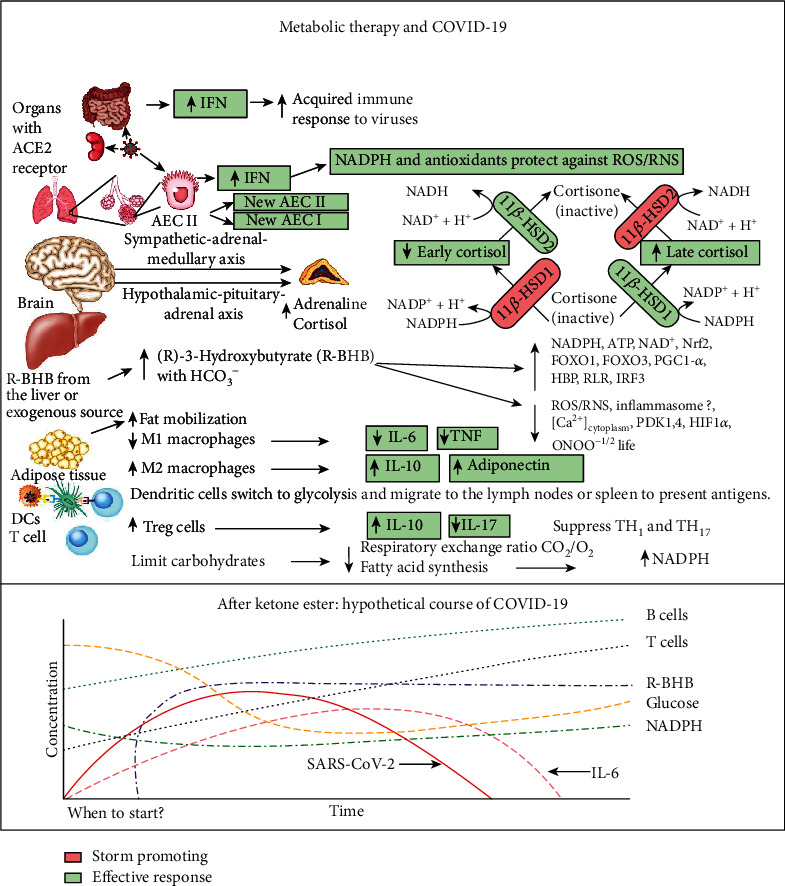
Proposed mechanisms and time course of SARS-CoV-2 infection when using a ketone-based metabolic therapy. Abbreviations: HBP: hexosamine biosynthesis pathway.

SARS-CoV-2 infects many cell types including type II alveolar epithelial cells (AEC II) in the lungs [4], where this leads to respiratory infection. AEC II either divide to maintain their levels or differentiate into AEC type I, which provide the surface area for the vast majority of gas exchange in the lungs [5]. Other important functions of AEC II include the secretion of surfactants, superoxide dismutase 3 (SOD3) [6], and type I (*α*/*β*) and type III (*λ*) interferons [7] to protect airway function. Due to these functions, AEC II have high energy requirements and rely heavily on fatty acid oxidation for energy production [8]. The partial loss of these functions during infection facilitates viral spread and disrupts the immune response and tissue repair. Nearly all nucleated cells, including AEC II, can recognize the presence of viruses and initiate an innate immune response to recruit phagocytic cells to the infection. RNA viruses such as SARS-CoV-2 are primarily recognized by cytosolic retinoic acid-inducible gene I-like receptors (RLRs), RIG-1, and melanoma differentiation-associated gene 5 (MDA5). The endosomal toll-like receptors (TLRs), TLR7/8 and TLR3, also play a role [9]. Excessive signaling through these endosomal TLRs can cause inflammatory pathology [10]. In a cytokine storm, the number of phagocytic cells, including macrophages and neutrophils, increases along with the levels of proinflammatory cytokines, while the numbers of B and T lymphocytes, mediators of the adaptive immune response, decline [11]. This results in a failure to clear the virus and facilitates a runaway positive feedback loop that increases the numbers of cytokine-secreting innate immune cells. This cytokine storm is emerging as a major contributor to acute respiratory distress syndrome (ARDS), multiple organ dysfunction, and patient death in COVID-19 [12, 13].  Figure 1 summarizes the molecular pathologies that occur during SARS-CoV-2 infection that lead to a cytokine storm and ARDS, while Figure 2 summarizes how metabolic therapy with ketone ester and a moderately high-fat diet may intervene in the disease process to protect against pathology.

### 1.2. Metabolic Therapy

**Figure 3:**
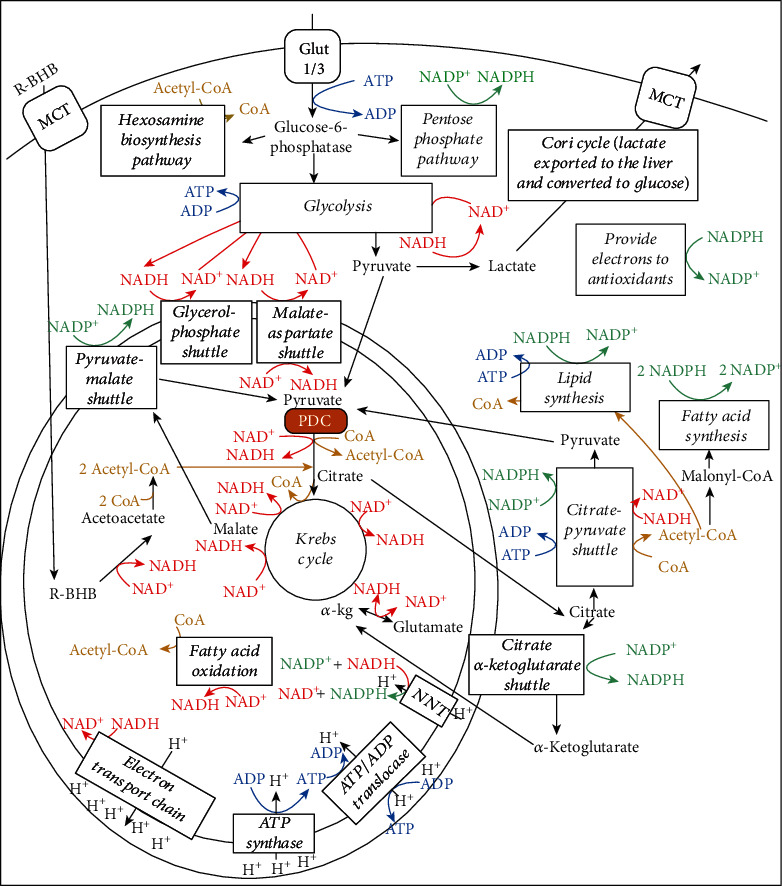
The major pathways of central metabolism and reactions that alter the ratios of coenzyme couples are shown. The mitochondrial matrix and the cytoplasm have independent coenzyme couple ratios. ATP synthesized in the mitochondrial matrix is exported to the cytoplasm in exchange for ADP by the adenine nucleotide translocase present in the inner mitochondrial membrane. Acetyl-CoA synthesized in the mitochondrial matrix must also be exported to the cytoplasm to provide two carbon units for fatty acid synthesis and protein acetylation. However, acetyl-CoA cannot cross the mitochondrial inner membrane. Therefore, the transfer of acetyl units across the inner mitochondrial membrane is accomplished using the citrate-pyruvate shuttle or the citrate-malate shuttle. These shuttles alter coenzyme levels and use inner membrane carrier proteins for the transport of citrate, pyruvate, and malate. The net result of the citrate-pyruvate shuttle on coenzyme levels is the use of energy from ATP hydrolysis and NADH oxidation to reduce NADP^+^ to NADPH. There is also a citrate-alpha-ketoglutarate shuttle system that has the net effect of using ATP hydrolysis to increase NADPH in the cytoplasm instead of increasing NADH or NADPH in the mitochondrial matrix. Synthesizing fatty acids in the cytoplasm and reducing antioxidants require NADPH. The malate-aspartate shuttle transfers reducing equivalents from NADH between the cytoplasm and the mitochondrial matrix. The glycerol 3-phosphate shuttle transfers reducing equivalents from cytoplasmic NADH to the mitochondrial ETC. Glucose is catabolized by three pathways including the hexosamine biosynthesis pathway that synthesizes uridine diphosphate (UDP) N-acetylglucosamine, glycolysis that reduces NAD^+^ to NADH and synthesizes ATP from ADP and P*_i_*, and the pentose phosphate pathway (PPP) that reduces NADP^+^ to NADPH and synthesizes ribose sugars for nucleotide synthesis. When the pyruvate dehydrogenase complex (PDC) is inhibited, lactate is synthesized from pyruvate to recycle NAD^+^ from NADH so glycolysis can continue. However, the lactate is exported from the cell and may contribute to lactic acidosis and multiorgan failure [20]. R-BHB decreases the reliance of cells on glycolysis leading to reduced cellular lactate export. Abbreviations: Glut1 and Glut3: glucose transporters 1 and 3; MCT: monocarboxylate transporter; NNT: nicotinamide nucleotide transhydrogenase; PDC: pyruvate dehydrogenase complex.

Central metabolism is controlled by four major nucleotide coenzyme couples: ADP/ATP, NAD^+^/NADH, NADP^+^/NADPH, and acetyl-CoA/CoA [14]. The prominent role these couples play in central metabolism is highlighted in Figure 3. Metabolic therapy aimed at restoring these ratios is often used as an adjunct to more targeted therapies [15]. The ketogenic diet as a treatment for childhood epilepsy has drawn focus to (R)-beta-hydroxybutyrate (R-BHB) as a metabolic therapy. Recently, exogenous ketones, which are various formulations of BHB, acetoacetate, or their precursors, have made it possible to raise blood R-BHB levels and alter the ratios of the controlling coenzyme couples without implementing a ketogenic diet [16]. (R)-3-Hydroxybutyl (R)-3-hydroxybutyrate, a type of ketone ester, is one of the several forms of exogenous ketones that increase systemic R-BHB levels. R-BHB-derived metabolites restore flux through the citric acid (Krebs) cycle and oxidative phosphorylation when viral-induced changes in enzyme activity prevent glucose [17] or fatty acids [18, 19] from fueling these pathways. Increasing R-BHB levels has been shown to normalize ADP/ATP, NAD^+^/NADH, NADP^+^/NADPH, and acetyl-CoA/CoA ratios in diseased tissue [16]. R-BHB has multiple anti-inflammatory signaling roles and functions as an epigenetic modifier to stimulate a program of gene expression that alters metabolism to restore cellular redox function. The focus of metabolic therapy is on the restoration of the coenzyme ratios that largely control metabolic flux through central metabolic pathways.

#### 1.2.1. Ketone Ester Consumption Blunts Decreased Immune Function in Humans

In a study of blood cytokine levels in well-trained cyclists who compete in multiday races, the levels of TNF-*α*, IL-6, IL-2, and IFN-*γ* were raised following intense exercise, indicating increased inflammation, whereas the level of IL-1*β* was unchanged [21]. On the last day of an eighteen-day trial, cyclists given daily ketone ester (R)-3-hydroxybutyl (R)-3-hydroxybutyrate showed a 15% higher mean power output and 25% increase in the CD4^+^/CD8^+^ (T helper cells/cytotoxic T cells) ratio than controls [22]. An increased CD4^+^/CD8^+^ ratio is associated with increased immune function [23], and this ratio declines with aging as immune system function declines [24].

#### 1.2.2. Ketone Ester Treatment Blunts the Cytokine Storm Induced by Ionizing Radiation in Model Systems

The same ketone ester used in the cycling studies has also been used in radiation mitigation studies. Cytokines are central to the pathophysiology of COVID-19; while some are beneficial, others are detrimental (IL-1*β*, IL-6, and TNF-*α*), at least in the context of the cytokine storm [25–28]. Exposure to acute doses of radiation results in tissue damage and an activation of cytokine cascades [26]. Several pharmaceutical approaches are being studied to prevent or decrease radiation-induced tissue damage and the cascade of harmful cytokines [29]. There is interest in using this radiation countermeasure strategy as a model for a viral-induced cytokine storm. IR has been shown to increase the expression of the following cytokines and growth factors including IL-4, IL-5, IL-10 [30], TGF-*β*, IL-12, IL-18 [31], type I interferons, IL-1*α*, IL-1*β*, IL-6, GM-CSF, and TNF-*α* [32, 33]. Maximal cytokine production occurs between 4 and 24 hours following exposure to short-term radiation [34, 35]. The balance of proinflammatory and anti-inflammatory cytokines synthesized is critical in the determination of outcomes [36] with several factors altering the profiles of the cytokines produced including the specific animal species and tissue studied, the magnitude of the radiation received, and whether whole animals, portions of animals, or only cells were exposed [30–33]. Chronic exposure to very low-dose nonacute radiation can induce hormesis and alter the levels of several cytokines to improve tissue responses [30, 35]. Further studies would be needed to determine the mechanism of these acute versus subacute radiation cytokine responses. Human polymorphisms in cytokine genes have been shown to be responsible for the differences in the extent of pathology that occurs following radiation damage [31]. Limited IR studies with acute radiation have demonstrated that ketone ester was able to decrease chromosomal damage in mice and increase survival in cells [37]. Ongoing studies are directly measuring the effects of ketone ester on animal survival following radiation and the effects on the radiation-induced cytokine storm. There are several other therapies being tested against a radiation-induced cytokine storm that could be considered for the treatment of COVID-19 as well [38].

### 1.3. Cytokines and Cytokine Receptors Are Also Promising Targets to Treat a Cytokine Storm

Several planned or recently initiated studies are targeting cytokines or their receptors in an attempt to blunt the cytokine storm of COVID-19. For example, the IL-6 receptor monoclonal antibody (mAb) antagonist tocilizumab has been identified as a strong candidate for treatment [39]. Other potential treatments to limit cytokine signaling include sarilumab (IL-6R mAb antagonist), anakinra (IL-1R recombinant protein antagonist) [40], and emapalumab (IFN-*γ* mAb). Some cytokines, such as type I interferons, may be beneficial to reduce the cytokine storm. SARS-CoV-2 was shown to be quite susceptible to treatment with type I interferons in vitro [41]. IL-7, which protects lymphocyte function, has been proposed as a therapy to treat lymphopenia that contributes to the cytokine storm [42]. Therefore, clinical studies to determine the effects of metabolic therapy with exogenous ketones in combination with one of these more targeted therapies should be considered for patients with severe SARS-CoV-2 infection.

## 2. R-BHB Decreases ROS/RNS Levels as a Mechanism to Blunt the Cytokine Storm

### 2.1. Increased ROS Levels Stimulate Inflammasome Activity and Cytokine Production

Reactive oxygen species (ROS) and reactive nitrogen species (RNS) levels increase in the lungs by at least two different mechanisms during the viral-induced cytokine storm. First, viral RNA binding to TLRs leads to decreased expression of mitochondrial electron transport chain (ETC) genes, which increases mitochondrial superoxide production [43–45]. Second, phagocytic cells are recruited to the lungs and, together with resident lung phagocytes, are activated through TLR7 and RIG-1 to increase NADPH oxidase 2 (NOX2) activity [46, 47], increasing the production of both intracellular and extracellular ROS and RNS to kill pathogens. However, host cells can be damaged as a byproduct of this response, especially in a cytokine storm. A review on the redox biology of respiratory viral infections has recently been published [48]. The increased ROS production from virus-induced TLR signaling [47], RIG-1 signaling [46], and altered mitochondrial function [49] leads to the activation of several transcriptional regulators such as NF-*κ*B, IFN-regulatory factor 3 (IRF3), and STAT1 to increase the production of cytokines, including TNF-*α*, IL-6, and IL-8, from AEC and macrophages [50]. The enhanced NF-*κ*B activity also leads to the activation of the NLRP3 inflammasome that increases IL-1*β* and IL-18 production [51]. The transcription of NF-*κ*B is a necessary step in the two-stage model of NLRP3 activation [52].

### 2.2. Secreted SOD3 and Catalase and Exogenous ROS Scavengers Protect against Extracellular ROS and Cytokine Storm

In the lungs, catalase and extracellular superoxide dismutase 3 (SOD3) [6] are synthesized at high levels by AEC II [53, 54]. In addition to its normal peroxisomal localization, catalase is secreted to the extracellular space by alveolar macrophages [55, 56] through a mechanism that is distinct from the classical secretory pathway [56, 57]. Older COVID-19 patients, who have a higher risk of mortality from the disease [58], were shown to express much less SOD3 from their AEC II than younger patients [59], suggesting an important role of SOD3 in protecting against the cytokine storm. These antioxidant enzymes reduce the concentration of toxic superoxide and hydrogen peroxide in extracellular fluids preventing oxidative damage to extracellular structures. In this regard, NOX2 has been shown to synthesize superoxide and release it into the luminal extracellular space mostly from AEC I [9, 60, 61]. Consistent with its important antioxidant function, polymorphisms in SOD3 are associated with reduced lung function and chronic obstructive pulmonary disease (COPD) [62].

Exogenous administration of catalase has been shown to mitigate respiratory viral infections. Intranasal catalase protected against respiratory syncytial virus (RSV) infection [63], a virus that can induce a cytokine storm [64]. Catalase treatment led to a significant reduction in the levels of the cytokines IL-1*α*, TNF-*α*, and IL-9 and the chemokines CSCL1, CCL2, and CCL5 [63]. During the early stages of other types of respiratory infections, increased ROS activates the nuclear factor erythroid 2-related factor 2 (NFE2L2 commonly called Nrf2) transcriptional regulator to induce antioxidant genes such as SOD3 and catalase to protect against the ROS-induced proinflammatory gene expression and subsequent cytokine storm. However, RSV infection leads to the proteolytic degradation of Nrf2, preventing the protective antioxidant response [6] to facilitate the cytokine storm. In addition, catalase can be inactivated by high levels of ROS and RNS in the lungs [65]. Influenza A virus (IAV) is another virus that induces a cytokine storm [66]. In mice infected with IAV, intranasal administration of the mitochondrial-targeted antioxidant, MitoTEMPO, quenched ETC-derived ROS in the lungs to decrease proinflammatory gene expression, cytokine storm, and consequent mortality [67]. A similar study found that intranasal administration of a NOX2 inhibitor had similar protective effects against IAV infection in mice [68, 69]. Therefore, both mitochondria- and NADPH oxidase-mediated increases in ROS contribute to IAV mortality. The role of NADPH oxidase in respiratory virus infections has been reviewed [9].

### 2.3. ROS and RNS Are Formed by the Oxidative Burst in Phagocytes

**Figure 4:**
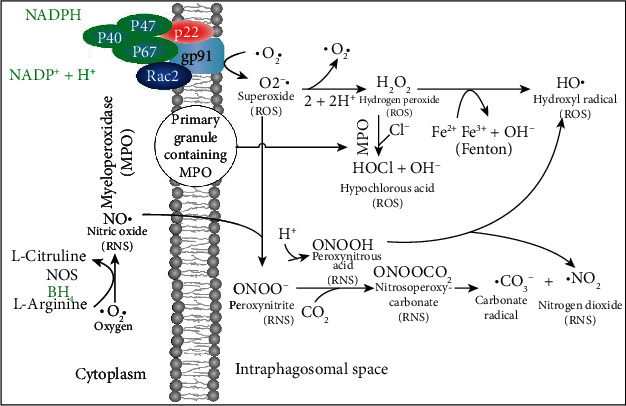
Phagocyte ROS and RNS metabolism. Most of the major forms of ROS and RNS are derived from superoxide or nitric oxide (NO).

**Figure 5:**
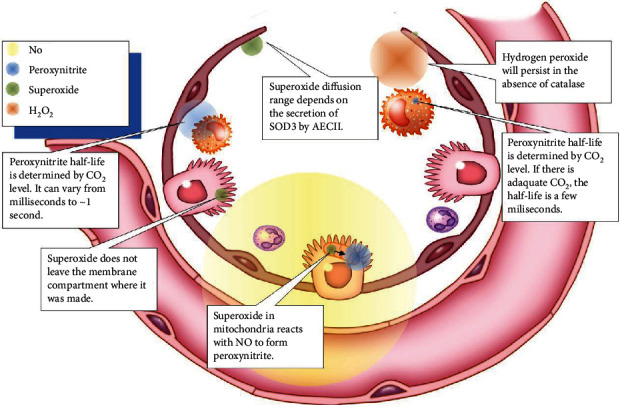
The half-lives and diffusion limits of ROS/RNS.

Phagocytes release proteolytic enzymes, ROS, and RNS into their phagosomes to mediate the killing of endocytosed pathogens. The phagosomal oxidative burst requires cytoplasmic NADPH as a coenzyme for membrane-bound NADPH oxidase to produce phagosomal superoxide (Figure 4). The iron-dependent metalloprotein myeloperoxidase (MPO) is a component of primary granules that fuse with the phagosome [70]. MPO catalyzes the synthesis of toxic hypochlorous acid from hydrogen peroxide and chloride ion. Two gases, nitric oxide and CO_2_, are critical for the synthesis of toxic carbonate radicals. For this to occur, first the superoxide radical must bind to the nitric oxide radical to form peroxynitrite, and then, peroxynitrite reacts with carbon dioxide to form nitrosoperoxycarbonate that degrades to the carbonate radical. Peroxynitrous acid, having a pKa of 6.8, forms physiologically when peroxynitrite binds to a proton and is a major source of hydroxyl radicals [71]. As CO_2_ levels increase, the half-life of peroxynitrite decreases from roughly a second down to several milliseconds [72]. The ROS and RNS that are produced during viral infection take on specific roles as sentinels, messengers, and oxidizing agents that determine the activity of many classes of proteins including transcription factors [71]. Hydroxyl radicals are short lived and function primarily as oxidizing agents. Superoxide is negatively charged and does not diffuse directly across lipid bilayers, but it has been shown to be transported by proteinaceous channels from the mitochondria to the cytoplasm [73, 74]. Hydrogen peroxide is transported by aquaporins across cellular membranes. The concentration of hydrogen peroxide in the cytoplasm is generally indicative of the health of mitochondria, but transient increases can be the result of signaling events. Nitric oxide is a free radical that passes through membranes and can potentially signal to nearby cells in its relatively short half-life. Peroxynitrous acid can also cross cellular membranes. The half-lives and diffusion limits of different types of ROS are shown in Figure 5. The importance of this diffusion of ROS and RNS between cells is that cells that lack the receptor for the virus can attempt to mount an appropriate response to the infection.

### 2.4. ROS/RNS Affects the Nucleotide Coenzyme Couples

Virus-induced ROS production and cytokine storm induce energy dysfunction and redox imbalance in host cells in the lungs by altering the ADP/ATP, NAD^+^/NADH, and NADP^+^/NADPH ratios that control central metabolism.

1. ROS produced by the mitochondrial ETC damages proximal ETC proteins resulting in decreased electron flux, which increases the cellular ADP/ATP (less ATP)
2. The decreased ETC flux also decreases the cellular NAD^+^/NADH (more NADH) as the rate of NADH hydrolysis by ETC complex I slows
3. The increased superoxide produced from the ETC is converted by superoxide dismutase 1 (SOD1) and SOD2 to hydrogen peroxide. H_2_O_2_ is detoxified by glutathione peroxidase through the conversion of reduced glutathione (GSH) to glutathione disulfide (GSSG). Increased activity of NADPH-dependent glutathione reductase is needed to recycle GSSG to GSH leading to an increased cellular NADP^+^/NADPH ratio (less NADPH). In a parallel pathway yielding the same result, hydrogen peroxide is detoxified by peroxiredoxins. The oxidized peroxiredoxins are then reduced by thioredoxins, and lastly, the oxidized thioredoxins are reduced by thioredoxin reductase using the reducing power of NADPH leading to an increased cellular NADP^+^/NADPH ratio

The ADP/ATP, NAD^+^/NADH, and NADP^+^/NADPH couples control hundreds of cellular reactions. When these levels are altered in cells during a cytokine storm, the cells can no longer effectively perform their primary functions leading to cell dysfunction and death and to pathologies such as ARDS.

## 3. Importance of Energy Metabolism in Blunting the Cytokine Storm

In immune cells, transitioning from an inactive state to an inflammatory and then to a postinflammatory state is accompanied by metabolic reprogramming. This assures that cells have adequate energy and redox potential to perform their new roles, including entering the cell cycle for propagation, performing an oxidative burst, or undergoing regulated apoptosis rather than necrosis. The mitochondrial PDC is well positioned to reprogram metabolism as it is the gatekeeper of carbohydrate flux into mitochondria as well as a major regulator of cellular NAD^+^/NADH. When it is active, it reduces mitochondrial NAD^+^, and when it is inhibited, it redirects pyruvate metabolism to the cytoplasm where lactate dehydrogenase reduces pyruvate by oxidizing NADH. PDC activity, by modulating the NAD^+^/NADH ratio, also affects the flux through glycolysis and the mitochondrial citric acid cycle, fatty acid oxidation, and oxidative phosphorylation.

### 3.1. Viral Infection Leads to Mitochondrial ETC Dysfunction and Decreased Energy Metabolism

The innate immune response to SARS-CoV-2 begins with the cellular production of the type I interferons *α* and *β* from plasmacytoid dendritic cells, macrophages, and AEC II. Viral double-stranded RNA replication intermediates stimulate TLR3 (toll-like receptor 3) leading to decreased ETC complex I gene expression [75], decreased ETC complex I activity, and increased ETC-mediated and NOX2-mediated ROS production [43–45], likely through type I interferon signaling [76], which increases proinflammatory cytokine production. In this regard, treatment with TNF-*α* was shown to downregulate the expression of peroxisome proliferator-activated receptor-*γ* coactivator-1*α* (PGC-1*α*), a master regulator of mitochondrial gene expression [77], decreasing mitochondrial ETC function and oxygen consumption in mouse lung AEC [8]. Downregulation of PGC-1*α* would likely increase ROS production as PGC-1*α* also induces antioxidant enzymes such as SOD2 and catalase [78]. Unexpectedly, peripheral blood mononuclear cells (PBMCs) from SARS patients were shown to have increased expression of mitochondrial-encoded subunits of the ETC [79], which could lead to increased ROS production due to incompletely formed ETC complexes when the nuclear-encoded subunits are downregulated. SARS-CoV-2 RNA has been shown to localize to mitochondria [80, 81], likely to attempt to hide from cellular immune surveillance systems. This could partly explain how SARS-CoV-type coronaviruses have been shown to be quite effective in blocking the type I interferon *β* response during the initial stages of the infection [82, 83].

### 3.2. Targeting the Viral-Induced Decrease in PDC Activity

In mice infected with IAV, ATP levels greatly decreased and the level of a negative regulator of PDC, pyruvate dehydrogenase kinase 4 (PDK4), increased substantially. Administration of diisopropylamine dichloroacetate (DADA), an inhibitor of PDK4, significantly delayed mortality from the infection [17]. However, the infection led to severe anorexia, which also increases PDK4 levels in some tissues such as those in the muscle [84] and liver [85] due to increased FOXO1, PPAR-*α*, and glucocorticoid receptor (GR) [86] transcriptional activity. This inactivation of PDC during starvation likely evolved to save glucose and lactate (which is converted back into glucose in the liver as part of the Cori cycle) for neurons, which do not efficiently oxidize fatty acids. The full extent to which the virus directly upregulated PDK4 levels is unknown. The levels of the proinflammatory cytokines TNF-*α*, IL-6, and IL-1*β* were shown to increase following IAV infection [17]. As stated above, a TLR3- and type I interferon-dependent response to viral RNA has been shown to reduce the expression of four subunits of mitochondrial ETC complex I [87]. This likely contributes to decreased ATP production. DADA administration significantly increased pyruvate dehydrogenase (PDH) activity and ATP levels in the skeletal muscles, heart, lungs, and liver and tended to normalize plasma levels of glucose, lactate, free fatty acids, and R-BHB [17]. DADA administration also suppressed the IAV-induced increase in IL-6, IL-2, IFN-*α*, TNF-*α*, and IFN-*γ* levels, but not that in IFN-*β* or IL-1*β* [17]. PDK inhibitors have also been shown to have protective anti-inflammatory effects. This may partly result from their effects on T lymphocytes, as proinflammatory Th17 cells have high levels of PDK1 and show primarily glycolytic metabolism, while anti-inflammatory Th1 and Treg cells have low PDK1 levels and show primarily oxidative metabolism. Knockdown of PDK1 suppressed Th17 cells and increased Treg cell numbers to restore immune function in mice with experimental autoimmune encephalomyelitis [88].

In another study of IAV infection in mice, glucose administration during the period of anorexia following IAV infection was found to decrease the mortality rate [89]. Glucose administration likely stimulated the insulin receptor-AKT signaling pathway to decrease FOXO1 activation to blunt the increase in PDK4 levels resulting from anorexia to maintain energy generation [86]. Other activators of mitochondrial energy metabolism, such as the peroxisome proliferation-activated receptor-gamma (PPAR-*γ*) agonist pioglitazone or rosiglitazone and the AMP kinase activator, AICAR, have also been shown to protect mouse mortality from IAV infection [90]. These compounds are all known to decrease PDK4 levels in the muscle and liver [91]. Therefore, FOXO1 hyperactivation may be a pathological event in mouse IAV infection as it is induced by both increased ROS levels [92] and the anorexia that occurs following IAV infection. A mechanism through which antioxidant administration protects against viral infection may be through preventing FOXO1 induction of PDK4. The use of PPAR-*γ* agonists to treat the cytokine storm in COVID-19 has been reviewed [93].

**Figure 6:**
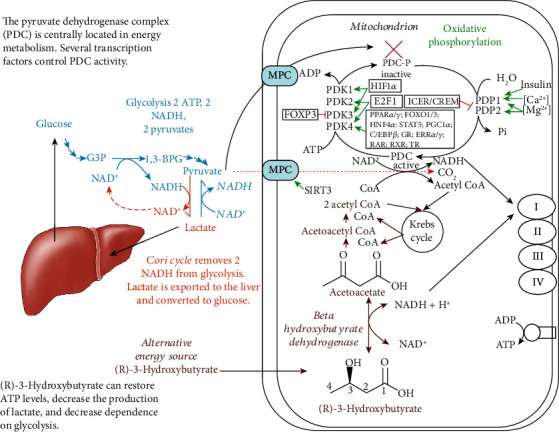
Restoring PDC activity or metabolizing R-BHB and fatty acids to bypass the inhibited PDC activity that occurs following viral infection is central to beneficial energy reprogramming. Most transcription factor names are taken from the *Encyclopedia of Signaling Molecules* [97, 98]. Abbreviations: 1,3-BPG: 1,3-bisphosphoglyceric acid; C/EBP*β*: CCAAT-enhancer-binding protein *β*; G3P: glyceraldehyde 3-phosphate; E2F1: E2F transcription factor 1; ERR*α* and ERR*γ*: estrogen-related receptor alpha and gamma; GR: glucocorticoid receptor; HNF4*α*: hepatic nuclear factor 4 alpha; ICER/CREM: inducible cAMP early repressor/cAMP-responsive element modulator; MPC: mitochondrial pyruvate carrier; PDC: pyruvate dehydrogenase complex; PGC1*α*: PPARG coactivator 1 alpha; PPAR*α* and PPAR*γ*: peroxisome proliferator-activated receptor alpha and gamma; RAR: retinoic acid receptor; RXR: retinoic x receptor; SIRT3: sirtuin 3; STAT5: signal transducer and activator of transcription 5; TR: thyroid hormone receptor.

Figure 3 shows the central role of PDC at the gateway between glycolytic and citric acid cycle metabolism.  Figure 6 shows the multiple transcription factors that control the expression of the kinases and phosphatases that regulate PDC activity as well as controlling the expression of the three enzymes that comprise the PDC.  Figure 6 also shows that R-BHB is catabolized into two acetyl-CoA molecules that enter the citric acid cycle and bypass PDC inhibition. An NADH, which fuels complex I of the ETC, is also generated during R-BHB oxidation to acetoacetate. R-BHB has also been shown to increase PGC-1*α* levels [94] and mitochondrial fusion [95, 96], which are known to increase mitochondrial energy generation. Therefore, ketone body catabolism is a substantial source of ATP when the cytokine storm leads to the block of the mitochondrial oxidation of carbohydrate catabolites.

### 3.3. Viral-Induced Ca^2+^ Dysregulation May Lead to ATP Decline, Osmotic Imbalance, Edema, and Restricted Lung Volumes

**Figure 7:**
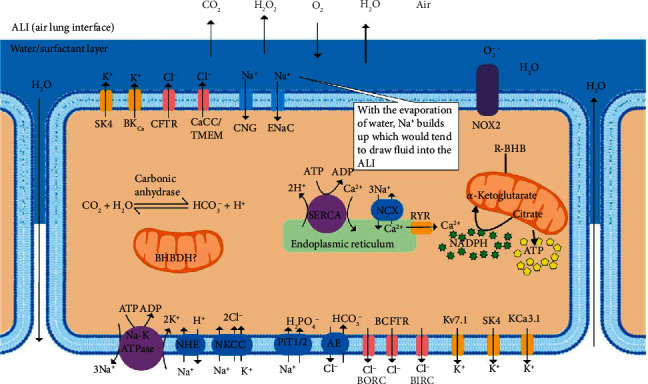
Maintaining ATP levels during respiratory viral infection is the key to maintain proper ion distribution to avoid edema. The active and passive cotransporters and channels maintain the ion gradients between AEC and extracellular fluids. The apical ligand-gated channels function to maintain appropriate osmotic pressure to provide sufficient fluid in the airway without causing edema. AEC I express superoxide-activated ENaC Na^+^ channels and NOX2 that regulates them. Abbreviations: 3Na^+^/2K^+^-ATPase: 3 sodium 2 potassium ATPase; AE: anion exchange chloride bicarbonate exchanger; BCFTR: basolateral cystic fibrosis transmembrane conductance regulator-like channel; BHBDH: *β*-hydroxybutyrate dehydrogenase; BIRC: basolateral inward rectifying channel; BK_Ca_: large-conductance Ca^2+^- and voltage-gated big K^+^ channel; BORC: basolateral outward rectifying channel; CACC/TMEM: calcium-activated chloride channels/transmembrane protein; CFTR: cystic fibrosis transmembrane conductance regulator; CNG: cyclic nucleotide-gated ion channel; ENaC: epithelial sodium channel; KCa3.1: calcium-activated potassium channel; Kv7.1: voltage-dependent potassium channel; NCX: sodium calcium exchanger; NHE: sodium-hydrogen exchanger; NKCC: sodium potassium chloride cotransporter; NOX2: NADPH oxidase 2; Pit1/2: sodium-dependent phosphate transporters 1 and 2; RYR: ryanodine receptor calcium-induced Ca^2+^ channel; SERCA: sarcoplasmic/endoplasmic reticulum Ca^2+^-ATPase; SK4: SK4 calcium-activated potassium channel.

There are nine major inorganic ions found intracellularly and extracellularly, Na^+^, K^+^, Ca^2+^, Mg^2+^, H^+^, Cl^−^, HCO_3_^−^, H_2_PO_4_^2-^, and HPO_4_^−^, which regulate plasma membrane potential and osmotic balance [99]. ATP drives the ion pumps that provide the chemiosmotic potential to maintain the distribution of these ions (Figure 7), preventing edema. The major SARS-CoV-2 spike (S) protein binds to extracellular Ca^2+^ to facilitate viral fusion with host cells such as AEC II [100] and signals for the opening of plasma membrane Ca^2+^ channels through a protein kinase C-*α* signaling pathway, which may be triggered by ER stress [101]. The SARS-CoV-2 envelope (E) protein is a lipidated viroporin, which forms a cation-selective channel in the endoplasmic reticulum that releases Ca^2+^ [102]. Blocking the viral-induced increase in cellular Ca^2+^ levels with a chelator decreased infectivity 60-fold [100]. The increased cytoplasmic Ca^2+^ leads to the activation of the plasma membrane and ER Ca^2+^ pumps, depleting ATP levels. Increased cytoplasmic Ca^2+^ stimulates the uptake of Ca^2+^ into the mitochondrial matrix in a mitochondrial membrane potential-dependent manner. In the presence of high levels of ROS caused by viral infection, the rapid uptake of Ca^2+^ into the mitochondrial matrix may stimulate permeability transition pores to open in the inner membrane [103], which uncouple mitochondria leading to further energy depletion and cell death. However, mammals have evolved mechanisms to use the viral-induced increase in cytoplasmic Ca^2+^ as a signal to upregulate host defenses. An increase in the cytosolic Ca^2+^ concentration by these mechanisms contributes to the activation of the NLPR3 inflammasome and elevation of IL-1*β* and IL-18 [102]. Increased cellular Ca^2+^ levels in AEC II lead to increased mitochondrial ETC-derived ROS and increased ROS from NADPH oxidases DUOX1 and DUOX2, the most abundant isoforms in AEC, through Ca^2+^ binding to their EF-hand motifs [9]. DUOX2 is also upregulated at the gene expression level by the increased interferon-*β* and TNF-*α* produced in response to the respiratory viral infection [104, 105]. Hydrogen peroxide can diffuse from AEC into adjacent cells leading to oxidative damage, energy depletion, and osmotic imbalance. Increased cytoplasmic Ca^2+^ levels in airway myocytes induce constriction of the airways. Several protein kinase C (PKC) isoforms are activated by Ca^2+^, and their activation is an important contributor to bronchoconstriction [106, 107]. PKC directly targets and inhibits Kv7 K^+^ channels, which are important for the relaxation of airway smooth muscle [108]. Inhibition of Kv7 channels induces bronchial constriction [109], which can contribute to ARDS. The use of R-BHB as an energy source may blunt the cellular energy deficit, the increase in cytoplasmic Ca^2+^ levels, and the osmotic imbalance to improve lung function.

## 4. High-Fat Diet Decreases the Average Time on a Ventilator for Patients with Respiratory Failure or ARDS

High-fat, low-carbohydrate enteral feeding of patients with type II respiratory failure (the inability to expel CO_2_ at a normal rate) reduced the mean length of time on a ventilator by 40%, from 158 hours down to 96 hours, compared to patients on high-carbohydrate, low-fat enteral feeding [110]. Arterial blood CO_2_ levels, an indicator of patient respiratory distress, decreased to 18% for patients in the high-fat group at the time of weaning off the ventilator. The high-carbohydrate group had an even higher partial pressure of CO_2_ at weaning than at the onset of ventilation. A likely contributor to the difference observed between the groups was the amount of CO_2_ synthesized from metabolizing the different diets. The amount of CO_2_ synthesized for every molecule of oxygen consumed is defined as the respiratory exchange ratio (RER). The RERs for catabolism of fat, glucose, and R-BHB are 0.7, 1.0, and 0.88, respectively. A high-fat diet was also protective in a mouse model of ventilator-induced lung injury [111], a model of ARDS. A high-fat, low-carbohydrate diet supplemented with fish oil, gamma-linolenic acid, and antioxidants was also shown to decrease the time on a ventilator for patients with ARDS due to sepsis/pneumonia [112], trauma, or aspiration injury; the findings may be relevant for ARDS mediated by viral infection as well.

## 5. Either Ketogenic Diet or Glucose Administration Protects against IAV Infection in Mice

Due to the lack of published data on the effects of increased R-BHB levels on the human immune system during viral infection, results obtained from several studies of metabolic therapy on IAV-infected mice are described below. A short-term ketogenic diet was shown to protect mice from IAV infection, while racemic R- and S-1,3-butanediol (BD), an exogenous ketone precursor, supplemented to a normal chow diet did not [113]. A long-term ketogenic diet that was obesogenic was shown to adversely affect glucose tolerance and immune system function [114]. As described above, glucose gavage during IAV-induced anorexia decreased mouse mortality [89], while a more recent study showed that glucose metabolism through the hexosamine biosynthetic pathway stimulated a cytokine storm [89]. In the sections below, the results from these studies will be described in more detail and analyzed in an attempt to reconcile these findings. The rationale will also be discussed for the consumption of a moderately high-fat, moderate-carbohydrate, ketone ester-containing diet at the onset of viral infection,and transitioning to a moderately high-fat, low-carbohydrate, ketone ester-containing diet if the infection becomes severe to blunt the cytokine storm.

### 5.1. A Ketogenic Diet Decreases IAV Mortality in Mice by Activating a *γδ* T Cell Response

A recent study showed that mice placed on a ketogenic diet for seven days before infection had decreased mortality from IAV [113]. The ketogenic diet increased the number of protective IL-17-secreting *γδ* T cells in the lungs. Administration of the ketone precursor BD to mice on a chow diet did not protect the survival or lead to the recruitment of *γδ* T cells to the lungs in the mice infected with IAV, even though the R-BHB blood level was equivalent to that of the ketogenic diet. One point raised regarding the design of this study is that while dietary protein amount (% *w*/*w*) was uniform between control and KD mice [115], the micronutrient and fiber profiles were not [116]. While this did not likely impact the conclusions of the study, it should be a point of emphasis for future experiments.

BD administration may not have shown protection due to a lack of improvement of the cellular redox environment in the lungs that likely occurred during the ketogenic diet. During times of high R-BHB oxidation in the brain, the cytoplasmic NADP^+^/NADPH becomes more reduced and the cytoplasmic NAD^+^]/[NADH becomes more oxidized [16]. The ketogenic diet, which is very high in fat content, may have improved the cytoplasmic redox environment, in part, through inhibition of fatty acid synthesis, an NADPH-consuming pathway, by increasing levels of palmitoyl-CoA, an inhibitor of fatty acid synthase activity. So, the combination of increased R-BHB metabolism and decreased fatty acid synthesis may lead to a decrease in the cytoplasmic NADP^+^/NADPH ratio that may lead to the recruitment and enhanced function of *γδ* T cells in the lungs, which does not occur when R-BHB is catabolized when mice are fed a normal chow diet. A high-fat but nonketogenic diet was shown to be ineffective in decreasing IAV-induced weight loss and mortality, even though it increased the recruitment of IL-17-secreting *γδ* T cells to the lungs. IL-17 binds to receptors on lung epithelial cells and possibly other lung cell types to increase the expression of IL-33. IL-33 secretion leads to the recruitment of type 2 innate lymphoid cells (ILC2s) to the lungs, where they play a role in regulating inflammation and barrier function by secreting the cytokines IL-5, IL-9, IL-13, and amphiregulin. In lung tissue, the ketogenic diet upregulated mitochondrial ETC gene expression and the expression of the OXCT1 gene encoding SCOT (succinyl-CoA:3-ketoacid CoA transferase), the rate-limiting enzyme for ketolysis, suggesting that R-BHB catabolism in the lungs plays an important role in the protective effects of the ketogenic diet against viral infection [113].

It is hypothesized that supplementation of ketone ester to mice on a moderately high-fat diet will lead both to the recruitment of *γδ* T cells to the lungs and to decreased mortality of IAV-infected mice. While one week of ketogenic diet prior to IAV infection was shown to be anti-inflammatory and decrease mouse mortality, three months of the ketogenic diet in the absence of IAV infection increased white adipose tissue (WAT) inflammation, decreased *γδ* T cell recruitment to the WAT, and led to obesity and glucose intolerance [114]. Therefore, current evidence from mouse studies where the animals were fed obesogenic ketogenic diets suggests that only short-term ketogenic diets will activate *γδ* T cells to boost immune function [114]. However, another research group identified a ketogenic diet of a different composition that was shown to induce weight loss in mice [117]. The dietary components responsible for the different effects on weight are currently unknown, although the leptogenic diet contained only half as much protein and used lard, butter, and vegetable oil as fat sources, while the obesogenic diet used hydrogenated soybean oil as the fat source [114, 117]. Future studies are needed to determine if a ketogenic diet that induces weight loss can provide long-term preservation of the protective *γδ* T cell response to provide long-term antiviral immunity.

It is also hypothesized that increased R-BHB levels improve cellular energy metabolism and redox status to enhance fatty acid beta-oxidation to overcome the metabolic inflexibility mediated by PDC inhibition. R-BHB is known to inhibit adipose tissue lipolysis [118], so a high-fat diet may be needed, along with exogenous ketones, to provide sufficient fatty acid beta-oxidation for increased metabolic flexibility to overcome a cytokine storm. The initiation of this diet in humans for COVID-19 poses challenges because it may take many days to adapt to a ketogenic diet to fully upregulate the expression of genes for ketogenesis, ketolysis, and fatty acid oxidation. Proinflammatory cytokines also inhibit ketogenesis [119]. In addition, starting a ketogenic diet, also called ketoinduction, may be accompanied by flu-like symptoms [120–122] that may limit its application to COVID-19 patients. However, studies could be performed to determine the ability of COVID-19 patients to tolerate a ketogenic diet supplemented with exogenous ketones, to attempt to decrease the early adverse effects of the diet that likely result from decreased energy production when glucose levels initially decline [122]. If well tolerated, further studies determining the ability of this diet to activate a protective immune response could follow.

Further experiments are needed to delineate the molecular mechanisms involved if R-BHB precursors such as ketone esters are to be used for the treatment of IAV and SARS-CoV-2 infections. To test the effectiveness of exogenous ketone administration on IAV infection, mice could be supplemented with or without a ketone ester and fed a moderately high-fat, moderate-carbohydrate diet or a moderately high-fat, low-carbohydrate diet or a control chow diet, and weight loss and mortality could be monitored following IAV infection. As explained in more detail below, glucose (from carbohydrate metabolism) stimulates important proinflammatory antiviral functions early in infection [123], but these proinflammatory actions also increase the cytokine storm late in infection [124]. So, it is unknown which of these effects will predominate to affect mortality in the presence of high R-BHB levels. It is hypothesized that the moderately high-fat, moderate-carbohydrate, ketone ester-containing diet will provide the most metabolic flexibility to stimulate host cell defense mechanisms. This flexibility should be able to preserve energy metabolism and redox status to boost immune function to decrease IAV titer in the lungs. However, the moderately high-fat, low-carbohydrate diet with ketone ester will likely show a stronger ability to blunt the cytokine storm, as increased glucose and insulin levels have recently been shown to block an important anti-inflammatory action of R-BHB [125].

### 5.2. Anorexia following IAV Infection in Mice Increases Mortality That Is Greatly Blunted by Gavage of Glucose, but Not Fat or Protein

As alluded to earlier, mice became anorexic following IAV infection and the anorexia contributed to their mortality, as glucose gavage was able to decrease the mortality. In that study, gavage of olive oil (fat) or casein (protein) did not decrease mortality [89]. The proinflammatory cytokines IL-1, IL-2, IL-6, IL-8, TNF-*α*, and IFN-*γ*, several of which increase following IAV infection, have been shown to suppress appetite [126]. So, it is likely that administering a ketogenic diet simply decreased cytokine levels, allowing for an increase in the amount of food consumed to decrease the mortality of the mice. Consistent with this interpretation, mice on the ketogenic diet lost less weight following infection than the chow-fed mice [113]. These results may be applicable to SARS-CoV-2 infection, as 40% of COVID-19 patients reported lack of appetite as a symptom [127]. A major research question that arises from these studies is whether a twice-daily isocaloric gavage of ketone ester starting on the day of infection, to mimic the protective effect of the twice-daily gavage of glucose [89], can protect mice fed a chow diet from IAV infection. This hypothesis is reasonable given that the protective ketogenic diet was composed of roughly 90% fat, 10% protein, and only 0.1% carbohydrate [113]. So, the carbohydrate content of the diet was likely too low to provide adequate glucose for protection, and gavage of fats or protein was unable to provide protection [89]. The ineffectiveness of the ketone precursor BD against IAV infection [113] suggests that ketone ester alone may be ineffective and that gavage of fat and ketone ester together as a cotherapy, to better mimic a ketogenic diet, may be needed for protection. Experiments probing possible additive or synergistic effects among glucose, ketone ester, and fats on increased survival during IAV infection in mice would provide valuable insights relevant to the protection against a cytokine storm in humans.

## 6. Glucose Metabolism through the Hexosamine Biosynthesis Pathway Protects against Viral Infection but Stimulates the Cytokine Storm

**Figure 8:**
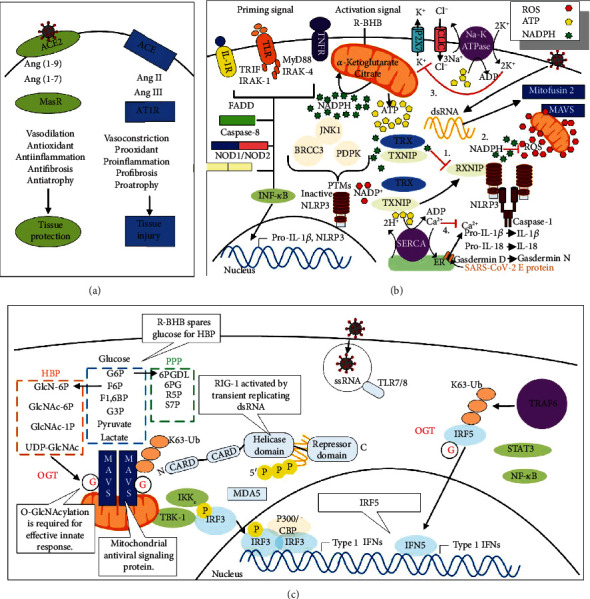
Proinflammatory signaling that occurs during viral infection and mechanisms through which R-BHB inhibits this signaling. (a) The ACE system. (b) The mechanism by which R-BHB inhibits the NLRP3 inflammasome is unknown. Four possible mechanisms are shown. (c) The hexosamine biosynthesis pathway is required to initiate an effective antiviral innate immune response, but its increased activity can also stimulate a cytokine storm. Abbreviations: 6PGDL: 6-phosphonoglocono-D-lactone; 6PG: 6-phosphogluconate; ACE2: angiotensin-converting enzyme 2; ACE: angiotensin-converting enzyme; ANG (1-9): angiotensin (1-9); ANG (1-7): angiotensin (1-7), a vasodilator; ANG II: angiotensin II, a vasoconstrictor; ANG III: angiotensin III, a metabolite of ANG II; BRCC3: Lys-63-specific deubiquitinase BRCC36; CARD: caspase recruitment domain; CLIC: chloride intracellular channel protein; F1,6BP: fructose 1,6-bisphosphate; F6P: fructose 6-phosphate; FADD: fas-associated protein with death domain; GlcNAc: G,N-acetylglucosamine; G3P: glyceraldehyde 3-phosphate; G6P: glucose 6-phosphate; GlcN-6P: glucosamine-6-phosphate; GlcNAc-6P: N-acetyl glucosamine-6-phosphate; GlcNAc-1p: N-acetyl glucosamine-1-phosphate; HBP: hexosamine biosynthesis pathway; IKK*_ε_*: I*κ*B kinase *ε*; IL-1R: interleukin-1 receptor; IRAK-1 and IRAK-4: interleukin-1 receptor kinases 1 and 4; IRF3 and IRF5: IFN-regulatory factors 3 and 5; JNK1: c-Jun N-terminal protein kinase 1; K63-Ub: K63-linked polyubiquitin binding; MasR: Mas receptor; MAVS: mitochondrial antiviral signaling protein; MDA5: melanoma differentiation-associated gene 5; MyD88: myeloid differentiation primary response 88; NF-*κ*B: nuclear factor kappa-light-chain-enhancer of activated B cells; NLRP3: NOD-, LRR-, and pyrin domain-containing protein 3; NOD1 and NOD2: nucleotide-binding oligomerization domain-containing proteins 1 and 2; OGT: O-linked N-acetylglucosamine (GlcNAc) transferase; P2X7: P2X purinoceptor 7; PDPK: phosphoinositide-dependent kinase-1; PPP: pentose phosphate pathway; PTMs: posttranslational modifications; R5P: ribose 5-phosphate; RIG-1: retinoic acid-inducible gene I; S7P: sedoheptulose 7-phosphate; SERCA: sarcoplasmic/endoplasmic reticulum Ca^2+^-ATPase; STAT3: signal transducer and activator of transcription 3; TBK-1: TANK-binding kinase 1; TLR: toll-like receptor; TNFR: tumor necrosis factor receptor; TRAF6: tumor necrosis factor receptor- (TNFR-) associated factor 6; TRIF: TIR domain-containing adapter-inducing interferon *β*; TRX: thioredoxin; TXNIP: thioredoxin-interacting protein; UDP-GlcNAc: uridine diphosphate N-acetylglucosamine.

So, how may glucose gavage of mice during viral-induced anorexia decrease mortality from IAV infection? Glucose has been shown to stimulate a proinflammatory antiviral response through increased flux through the hexosamine biosynthesis pathway. The increased flux increases levels of the pathway end product UDP N-acetylglucosamine (UDP GlcNAc), which increases the *O*-GlcNAcylation of the antiviral protein MAVS to increase its function (Figure 8(c)) [123]. The SARS virus synthesizes the NPS15 protein, which partially inhibits MAVS function to block host antiviral signaling [128]. In addition, viral nucleic acids and type I interferon signaling in macrophages lead to the increased expression of the glycolytic activator 6-phosphofructose-2-kinase and fructose-2,6-bisphosphatase (PFKFB3), which are required for the increased engulfment of viral-infected cells [129]. Surprisingly, mortality from IAV infection in mice has been linked with viral induction of an ER stress-induced apoptotic pathway in the brain [89]. Both glucose and R-BHB are important protective fuels for neurons, potentially reconciling findings of how either glucose or a ketogenic diet is protective. In addition, high glucose levels may compensate for the energy and redox crisis occurring as a result of viral-induced PDC inhibition by increasing flux through glycolysis for the synthesis of ATP and by increasing flux through the pentose phosphate pathway (PPP) for the synthesis of NADPH. Glucose flux through the hexosamine biosynthesis pathway has been shown to stimulate the cytokine storm during IAV infection in mice by increasing the *O*-GlcNAcylation of the transcriptional regulator IRF5, which increases its activity to stimulate proinflammatory cytokine production (Figure 8(c)) [124]. This may explain in part why people with diabetes who are infected with SARS-CoV-2 have a higher mortality rate [130]. Therefore, inhibitors of IRF5 or inhibitors of *O*-GlcNAcylation, such as OSMI-1, are potential treatments for the SARS-CoV-2 cytokine storm. Ketone ester treatment has been shown to decrease blood glucose levels [131, 132], which would likely decrease flux through the hexosamine biosynthesis pathway in immune cells to decrease cytokine production.

## 7. Molecular Mechanisms through Which R-BHB Inhibits Inflammation

Monocarboxylate transporters are expressed in AEC II [133], allowing the entry of R-BHB into the cytoplasm. However, these cells have low expression of ketolytic enzymes, so they may be unable to substantially catabolize the R-BHB produced from the consumption of exogenous ketones [134]. However, a ketogenic diet was shown to increase the expression of ketolytic genes in the lungs [113], so it is possible that these enzymes can be induced in AEC II and are responsible, in part, for the protective effects of the ketogenic diet. Even if R-BHB is not catabolized by AEC II, the presence of R-BHB in AEC II may still greatly protect these cells through signaling pathway activation, through enzyme inhibition, and through gene expression pattern alteration as described in detail below.

### 7.1. R-BHB Inhibition of the NLRP3 Inflammasome May Depend upon the Metabolic State of the Cell

R-BHB inhibits the NLRP3 inflammasome [135]. S-BHB, the enantiomer of R-BHB, was also effective, but not butyrate. The molecular target through which R-BHB and S-BHB inhibit the inflammasome is still unknown, but treatment decreased cellular K^+^ efflux and reduced inflammasome activator ASC (apoptosis-associated speck-like protein containing a CARD) oligomerization (Figure 8(b)). R-BHB-mediated inhibition of the inflammasome did not require ketone body catabolism, since siRNA of the ketolytic enzyme SCOT did not block inhibition. Inflammasome inhibition was also shown to be independent of the effects of R-BHB on GPR109A G-protein-coupled receptor (GPCR) signaling and histone acetylation. In addition to immune cells, several types of epithelial cells express the genes for a functional NLRP3 inflammasome; R-BHB likely protects AEC II from a cytokine storm in part through this mechanism [136]. Recently, high insulin or high glucose levels were shown to decrease R-BHB-mediated inhibition of the NLRP3 inflammasome in macrophages in vitro, and 2-deoxyglucose, a glycolysis inhibitor, was shown to potentiate NLRP3 inflammasome inhibition by R-BHB [125]. Therefore, the metabolic state of the cell appears to influence the effect of R-BHB on the NLRP3 inflammasome.

Somewhat surprisingly, a single dose of exogenous ketones was shown to increase inflammasome activation in LPS-stimulated blood cells and increase plasma IL-1*β* and IL-6 levels of healthy young people after a 10-hour overnight fast [137]. The mechanisms remain unknown, but increased levels of these proinflammatory markers may have been due to an R-BHB-mediated increase in NADPH levels stimulating NADPH oxidase activity to increase ROS levels and possibly also due to increased mitochondrial ROS production that occurs when R-BHB and glucose are oxidized simultaneously, as increased ROS stimulates NLRP3 inflammasome activity [138]. However, a follow-up study by the same group administering exogenous ketones to obese subjects found no difference in inflammasome activity and the levels of many proinflammatory markers but a slight decrease in IL-1*β* and TNF-*α* levels in the exogenous ketone-treated group [139]. In a study of well-trained cyclists, acute BD administration was shown to slightly increase interferon-gamma expression in PBMCs, while anti-inflammatory cytokine expression was unaltered [140]. Overall, the lack of NLRP3 inflammasome inhibition and the lack of strong anti-inflammatory effects of exogenous ketones in the above human studies are likely due to the metabolic state of the subjects when the exogenous ketones were administered. It is possible that the glucose levels and insulin levels were too high to allow R-BHB to inhibit the inflammasome [125].

SARS-CoV-2 infection can cause ketosis and ketoacidosis, and these patients with high blood R-BHB levels had longer hospitalization and an increased mortality rate [141]. Also, COVID-19 patients with type I or II diabetes mellitus (DM) have an increased risk of developing diabetic ketoacidosis (DKA), which contributes to mortality [142]. The molecular basis for these findings is not entirely clear as ketone bodies do not directly cause DKA. Recent findings, however, indicate that the increased glucose levels in mouse models of diabetes can decrease the expression of the ketolytic genes R-BHB dehydrogenase (BDH1) and OXCT1 in the heart [143]. If this also occurs in other tissues such as skeletal muscle, it would likely lead to increased blood ketone levels. Expressing an exogenous transgene to increase O-GlcNAcylation in the mice further decreased BDH1 levels demonstrating a role for the hexosamine biosynthetic pathway in this downregulation of ketolytic gene expression. The SCOT enzyme (OXCT1 gene product) was shown to be directly modified by O-GlcNAcylation. Therefore, administration of an O-GlcNAcylation inhibitor together with exogenous ketones to diabetic patients with COVID-19 may be beneficial to prevent decreased ketolysis and DKA.

Multiple factors may be contributing to acidosis in COVID-19 patients. Respiratory acidosis occurs due to a buildup of carbon dioxide in the body, while lactic acidosis occurs due to mitochondrial ETC dysfunction or PDC inhibition. R-BHB metabolism, unlike glucose metabolism, does not raise the levels of lactic acid and may even decrease acidosis by lowering the rate of glycolysis and lactic acid synthesis. Differential diagnosis and treatment of acidosis have been reviewed [144]. Consumption of a ketone ester has been shown to lower glycemic response in both healthy and obese people [131]. Fatty acid lipolysis in white adipose tissue is inhibited by ketones [118], so in most cases, exogenous ketones will inhibit the synthesis of endogenous ketones. Before insulin was available, a ketogenic diet that limited carbohydrates to ≤10 g/day was a commonly used effective therapy for type I diabetes [145]. During DKA, there are imbalances in the levels of glucagon and insulin and elevation of the stress hormones epinephrine, cortisol, and growth hormone. These changes can be triggered by a stressful event such as COVID-19. Therefore, care would need to be taken in administering exogenous ketones in a clinical trial for COVID-19. Coadministration of sodium bicarbonate may also be beneficial for diabetic COVID-19 patients to buffer changes in blood pH. In this regard, a recent study showed that cyclists administered ketone ester had a 20% decrease in blood bicarbonate levels and a slight decrease in blood pH, while blood R-BHB levels rose to 2-3 mM. Administering bicarbonate together with ketone ester prevented these alterations in the blood and increased blood R-BHB levels another 0.5-0.8 mM, and this resulted in a 5% increase in power output [146]. If diabetics are to be included in a COVID-19 trial testing the effects of exogenous ketones, the pH of arterial blood gas (ABG) and the blood levels of ketones would need to be monitored by experts in managing DKA to identify early-stage ketoacidosis so that interventions according to best practices [147] could be implemented. To avoid risks, patients with naturally high ketone levels should avoid exogenous ketones, so DKA may be an exclusion factor in trials. But ultimately, a clinical trial will likely be necessary to determine the effects of exogenous ketone consumption or a ketogenic diet on COVID-19 in both diabetic and nondiabetic patients.

The lack of protective effects of high R-BHB levels under certain metabolic conditions is likely due to the same underlying molecular mechanism that prevented supplementation with the ketone precursor BD from preventing mortality in IAV-infected mice [113]. A protective anti-inflammatory *γδ* T cell response was likely not initiated in these studies. In the studies with exogenous ketones, this was likely due to the acute nature of the exogenous ketone treatment and to the lack of the high-fat, low-carbohydrate diet that may be necessary to initiate this protective anti-inflammatory response. However, there are several other potential mechanisms that may have also prevented the acute ketone ester treatment from influencing the activation state of the inflammasome. For example, a 24-hour fast in human subjects has been shown to lead to NLRP3 inflammasome inactivation, due to increased mitochondrial NAD^+^/NADH activating the NAD^+^-dependent SIRT3 protein deacetylase to decrease ROS production [148]. Therefore, the 10-hour overnight fast may have led to a partial inhibition of NLRP3 inflammasome activity so that ketone ester treatment was unable to decrease the activity any further. It is also possible that at least five days of a moderately high-fat, low-carbohydrate diet with exogenous ketone treatment may be needed to show large anti-inflammatory effects, as it was shown to take five days to fully upregulate the activity of the fatty acid beta-oxidation system after initiating a ketogenic diet [149, 150].

### 7.2. R-BHB Functions as a Histone Deacetylase Inhibitor to Decrease Inflammation

**Figure 9:**
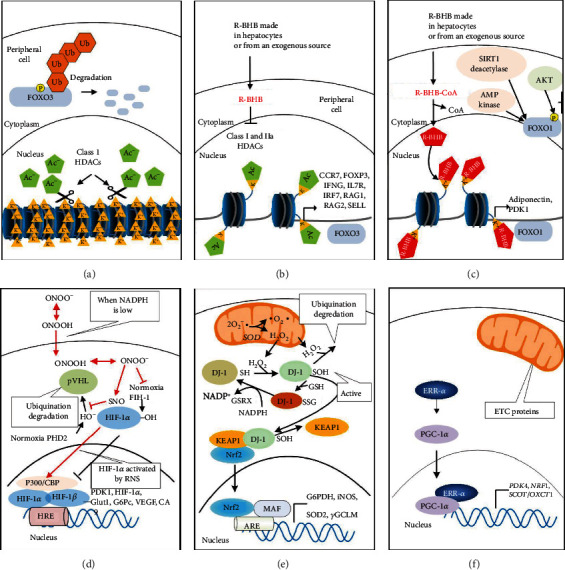
The effects of increased R-BHB levels on the activity of the transcription factors FOXO3a, FOXO1, HIF1-*α*, Nrf2, and PGC-1*α*. (a) DNA wrapped around histones with deacetylated lysines blocks the access of transcription factors, so HDAC function represses transcription. (b) R-BHB, by inhibiting class I HDACs, allows increased histone acetylation, relaxed chromatin, and FOXO3a expression. (c) Chromatin is also relaxed by *β*-hydroxybutyrylation at promoters such as that of FOXO1 to increase transcription. (d) HIF-1*α* is activated by RNS. Peroxynitrite S-nitrosylates HIF-1*α* to inhibit its von Hippel-Landau factor-induced ubiquitination and proteasomal degradation. This stabilizes HIF-1*α* leading to increased PDK1 expression. This stabilization is likely inhibited by R-BHB, which increases NADPH to decrease ROS/RNS levels. (e) Nrf2 is activated by oxidized DJ-1, and this is regulated by the redox potential of NADP^+^/NADPH that controls the cellular antioxidant potential. The DJ-1 chaperone protein, which is activated by moderate levels of hydrogen peroxide, stimulates the release of Nrf2 from KEAP1 allowing Nrf2 to enter the nucleus and induce antioxidant response element gene expression. (f) PGC-1*α* is the master regulator of mitochondrial biogenesis. It is a coactivator of ERR-*α*, FOXO1, FOXO3a, PPARs, and nuclear respiratory factor 1 (NRF1). Abbreviations: Ac: acetate; ARE: antioxidant response element; CA9: carbonic anhydrase 9; CCR7: C-C chemokine receptor type 7; ERR-*α*: estrogen-related receptor alpha; ETC: electron transport chain; K: lysine; HDAC: histone deacetylase; FIH-1: factor inhibiting HIF-1; FOXO1: forkhead box O1; FOXO3: forkhead box O3; FOXP3: forkhead box P3; G6PC: glucose-6-phosphatase; G6PDH: glucose 6-phosphate dehydrogenase; *γ*GCLM: glutamate cysteine ligase modifier subunit; Glut1: glucose transporter 1; GSRX: glutathione reductase; HIF-1*α* and HIF-1*β*: hypoxia-inducible factor 1 alpha and beta; HRE: hypoxia response element; IFNG: interferon gamma gene; IL7R: interleukin-7 receptor; iNOS: inducible nitric oxide synthase; IRF7: interferon regulatory factor 7; KEAP1: Kelch-like ECH-associated protein 1; MAF: musculoaponeurotic fibrosarcoma; Nrf1: nuclear respiratory factor 1; Nrf2: nuclear factor erythroid 2-related factor 2 (NFE2L2); RAG1 and RAG2: recombination activating genes 1 and 2; ONOO^−^: peroxynitrite; ONOOH: peroxynitrous acid; P300/CBP: binding protein 300/CREB-binding protein; PDK1 and PDK4: pyruvate dehydrogenase kinases 1 and 4; PGC1-*α*: PPARG coactivator 1 alpha; PHD2: prolyl-hydroxylase domain protein 2; pVHL: von Hippel-Landau tumor suppressor protein; SCOT/OXCT1: succinyl-CoA-3-oxaloacid CoA transferase also known as 3-oxoacid CoA-transferase 1; SELL: selectin L; SOD2: superoxide dismutase 2; Ub: ubiquitin; VEGF: vascular endothelial growth factor.

R-BHB was shown to be a class I and IIa histone deacetylase (HDAC) inhibitor (K_*i*_ = 2 − 5 mM) that induced expression of several antioxidant genes and the transcriptional regulator FOXO3a (Figures 9(a) and 9(b)) [151]. Administration of the other HDAC inhibitors, butyrate or trichostatin A, showed anti-inflammatory effects on lung ILC2s, while adding both compounds together showed no additive benefit [152]. This suggested that HDAC inhibition is a protective mechanism through which R-BHB and the ketogenic diet prevent lung inflammation. There is a nuclear pool of PDC that contributes to acetyl-CoA synthesis for histone acetylation [153]. PDK1 also shows a partial nuclear localization [154], so the upregulation of PDK1 expression during viral infection could disrupt nuclear histone acetylation, which could be restored by HDAC inhibitors such as R-BHB.

In studies with macrophages, butyrate was shown to function as an HDAC inhibitor to decrease IL-6, IL-12, and nitric oxide levels, but not TNF-*α* or MCP-1 levels [155]. In a co-culture model of RAW264.7 macrophages and 3T3-L1 preadipocytes, addition of butyrate decreased the production of TNF-*α*, MCP-1, and IL-6 and decreased NF-*κ*B expression in the macrophages [156]. Another study found that HDAC inhibition decreases NF-*κ*B transcription, which may be responsible for the anti-inflammatory effects [157]. Therefore, increasing R-BHB levels will likely lead to similar anti-inflammatory effects on lung macrophages to dampen a cytokine storm, although butyrate has been reported to be a superior HDAC inhibitor compared to R-BHB in some cell types such as myotubes and endothelial cells [158]. This decreased efficacy of R-BHB as an HDAC inhibitor in some cell types may result from different rates of transport into the cell or into the mitochondrial matrix, different rates of R-BHB oxidation, or different endogenous nuclear histone acetyltransferase or HDAC activities. Dietary therapies that increase both butyrate and R-BHB levels may have additive anti-inflammatory effects [159]. The antibacterial effect of butyrate on intestinal macrophages was shown to be due to HDAC3 inhibition, not GPR109A signaling. HDAC3 inhibition led to a decreased rate of glycolysis and increased flux through the PPP increasing AMP levels and AMP kinase activity and decreasing mTOR activity to stimulate autophagy [160]. In the lung, butyrate inhibition of the class IIA HDAC, HDAC7, decreased bacterial-induced inflammation [161]. During infections, mitochondrial damage leads to the oxidation and release of the inner membrane phospholipid cardiolipin, leading to PPAR-gamma SUMOylation and recruitment of HDAC3 to the promoter of IL-10, an anti-inflammatory cytokine, to decrease gene expression. Gene expression of TNF-*α* was unaffected, so increased inflammation was observed. Butyrate administration increased IL-10 gene expression to normalize the level of inflammation [162]. Coronaviruses have been shown to increase the oxidation of phospholipids, which stimulate toll-like receptor 4 (TLR4) signaling on macrophages, leading to cytokine production and acute lung injury [163], so HDAC inhibition with R-BHB appears to be a viable treatment to decrease cytokine levels and inflammation.

### 7.3. R-BHB Binds to the GPR109A GPCR to Stimulate Anti-Inflammatory Signaling

The GPR109A (hydroxycarboxylic acid receptor 2 (HCA2), expressed from the HCAR2 gene) GPCR is bound and activated by R-BHB (EC_50_ of 0.7 mM [118]), S-BHB, or butyrate and is expressed in the lung and many types of epithelial cells, macrophages, neutrophils, and dendritic cells, but not in B or naïve T lymphocytes [164]. However, GPR109A was shown to play a role in the expansion of CD4^+^ and CD8^+^ T cells [165]. The expression pattern of GPR109A suggests that it could play a role in the protective effects of the ketogenic diet against IAV infection [113]. GPR109A has been shown to be activated by Zika virus infection and protect cells by inhibiting viral replication [166]. A major mechanism through which GPR109A signaling exerts its anti-inflammatory effects is through suppressing the activation of the transcriptional regulator nuclear factor-kappa B (NF-*κ*B) [167], required for the transcription and secretion of several proinflammatory cytokines [168].

Studies with GPR109A-knockout mice have identified GPR109A signaling as essential for the increase in thermogenesis induced by its ligands [169]. Consistent with this, GPR109A-knockout mice were obese, showing hepatic steatosis due to upregulation of enzymes of fatty acid synthesis (ACC1 and fatty acid synthase (FAS)) and downregulation of enzymes of fatty acid oxidation (CPT-1*α*). PPAR-*α*, the master regulator of ketogenesis, was decreased in the liver, while PPAR-*γ*, the master regulator of adipogenesis, was increased in WAT. So, it is likely that stimulation of GPR109A plays an important role in the induction of fatty acid beta-oxidation and weight loss induced by the ketogenic diet. Macrophages and dendritic cells from GPR109A-deficient mice were defective in inducing naïve T cells to differentiate into Treg cells and IL-10-producing T cells [170]. Lack of GPR109A also decreased the expression of IL-18 [170]. GPR109A signaling has been shown to be protective by activating the Nrf2 transcriptional regulator through an AMP kinase signaling pathway to decrease oxidative stress [171]. GPR109A was also shown to play an important role in maintaining epithelial barrier function during bacterial sepsis [172], so it may play a similar role during viral infection.

### 7.4. R-BHB May Stimulate the Expression of the LL-37 Antiviral Peptide and Protect It from Inactivation

Cathelicidins are a class of antimicrobial host defense peptides. LL-37 is one of two human cathelicidins and released by bronchial epithelial cells, macrophages, and neutrophils as part of the innate immune response against respiratory viral infections [173]. LL-37 has many functions, including binding to nucleic acids, strengthening the viral RNA-induced TLR3 signaling response to increase type I interferon production [174], stimulating inflammasome activation [175], and reducing viral load and virion release [176, 177]. The peptide has both proinflammatory and anti-inflammatory properties [178], but the anti-inflammatory properties may predominate in the lungs as LL-37 administration decreased the expression of the proinflammatory cytokines IL-8 and IL-6 and the chemokine CCL5 in response to respiratory viral infection [179]. Respiratory viral infection increases the expression of peptidyl arginine deiminase 2 (PAD2) in the lungs. This class of enzymes catalyzes the removal of a positively charged amino group from protein arginine to form citrulline, through a process called citrullination. LL-37 has five arginine residues essential for its antiviral function, which are targets of PAD2 function following the viral-induced increase in PAD2 expression in the lungs [179]. Increased levels of NADPH decrease the catalytic activity of peptidyl arginine deiminases to limit LL-37 citrullination [180, 181]. HDAC inhibitors such as butyrate have been shown to increase the expression of LL-37 [182] to decrease pathogen infection [183]. Therefore, R-BHB may mitigate respiratory virus infection both by increasing LL-37 levels and by increasing NADPH levels [16] that protect LL-37 from inactivation.

### 7.5. Cortisol Is Transiently Increased in Plasma by a Ketogenic Diet and Its Level in Tissues Is Regulated by Redox-Sensitive Coenzyme Ratios

Cortisol, an adrenal gland-secreted hormone, has anti-inflammatory properties through activation of the glucocorticoid receptors. Subjects on either a ketogenic diet [184, 185] or a severe calorie restriction diet [186] show transient increases in cortisol levels. In mice, seven days of ketogenic diet led to the transcriptional activation of targets of the glucocorticoid receptor [113]. If this transient increase in cortisol levels that occurs in humans also occurs in mice on the ketogenic diet, the increased cortisol levels may contribute to the blunting of the cytokine storm in animals that were fed the ketogenic diet for a week before IAV infection [113]. Mice that were fed the ketogenic diet for three months showed increased inflammation, which could have been in part due to the return of cortisol to baseline levels [114].

As may be expected due to the function of cortisol as a stress hormone, when healthy subjects were administered ketone ester in nonketogenic states, no change in cortisol levels was observed [22, 187]. In peripheral tissues, such as the lungs, the level of cortisol is regulated by the 11*β*-hydroxysteroid dehydrogenase (11*β*-HSD) system consisting of the two enzymes, 11*β*-HSD1 and 11*β*-HSD2. The conversion of cortisone to the active steroid hormone cortisol is catalyzed by NADPH-dependent 11*β*-HSD1 [188], while the reverse reaction that regenerates the precursor cortisone is catalyzed by the NAD^+^-dependent 11*β*-HSD2 enzyme (Figures 1 and 2). Therefore, the level of active cortisol in tissues is under tight control by the NAD^+^/NADH and NADP^+^/NADPH redox ratios. Ketone ester treatment has been shown to normalize these coenzyme ratios in diseased mouse tissue [16]. Corticosteroid hormone administration has been used in an attempt to blunt the cytokine storm in several human respiratory viral infections [28]. To be successful, this therapy must be administered at the appropriate time late in the infection cycle to allow the immune system to first mount a proper antiviral response. Since identifying the appropriate timeframe for treatment for different patients is challenging, glucocorticoid therapy has been largely unsuccessful and may have even contributed to detrimental patient effects when used to treat influenza infection [189]. However, emerging data suggest that short-term dexamethasone treatment may be beneficial for SARS-CoV-2 infection [190], and dexamethasone treatment was shown to decrease the mortality of patients with severe SARS-CoV-2 infection who were placed on a ventilator [191]. Increasing R-BHB levels, together with a moderately high-fat diet, may be able to stabilize nucleotide coenzyme ratios to allow virally infected tissue to increase endogenous cortisol levels at the appropriate time in the infection cycle to decrease inflammation and blunt the cytokine storm.

### 7.6. R-BHB May Blunt Renin-Angiotensin Proinflammatory Signaling through HDAC Inhibition

SARS-CoV-2 virions bind to angiotensin-converting enzyme 2 (ACE2) receptors [192, 193] on the surface of host cells, such as AEC II, as a first step in viral entry [194]. The level of ACE2 receptors decreases during aging and may also decrease due to endocytosis during SARS-CoV-2 infection [195, 196]. In this renin-angiotensin signaling system, renin catalyzes the conversion of angiotensinogen into angiotensin 1 (ANG I). Angiotensin-converting enzyme 1 (ACE1) then catalyzes the conversion of ANG I into angiotensin II (ANG II), which binds to the AT1R receptor leading to vasoconstriction and proinflammatory, prooxidative, and profibrotic effects leading to tissue injury. ACE2 is normally able to blunt these effects by cleaving ANG I and ANG II into peptides that bind to the AT2R and MasR receptors that signal for vasodilation and anti-inflammatory, antioxidative, and antifibrotic effects leading to tissue protection (Figure 8(a)) [197–201]. Increased levels of ANG II stimulate the synthesis of the proinflammatory cytokines IL-6, IFN-*γ*, TNF-*α*, and IL-1*β* [202], but also the anti-inflammatory cytokines TGF-*β*1 and IL-10, which may induce M2 macrophage polarization [203] and prevent the *γδ* T lymphocyte activation needed to initiate the antiviral immune response [204]. Butyrate and other HDAC inhibitors have been shown to decrease the expression of angiotensinogen, renin, and AT1R to block this proinflammatory signaling [205, 206]. Therefore, the use of exogenous ketones could lead to a balancing of signaling through the different arms of the renin-angiotensin system when proinflammatory signaling through ANG II predominates such as in aged individuals and subjects infected with SARS-CoV-2.

### 7.7. R-BHB Has Different Effects on Proinflammatory Cytokine Production in Different Cell Types

The effects of R-BHB on proinflammatory cytokine production in different cell types can vary greatly. For example, R-BHB, when given to isolated macrophages challenged with *Streptococcus uberis*, was shown to increase the expression of IL-1*β* and IL-10 and the chemokines CXCL2 and CCL5 but had no effect on the expression of TNF-*α* and TGF-*β* [207]. In another report using isolated M1 peritoneal macrophages, R-BHB was shown to decrease the expression of IL-15, but not IL-1*β*, TNF-*α*, or IL-6 [208]. In calf hepatocytes, R-BHB was shown to increase NF-*κ*B activity and the expression of IL-1*β*, TNF-*α*, and IL-6 [209], while ketosis had similar effects in the liver of cows [210]. The absence of ketolytic enzymes and presence of ketogenic enzymes in the liver may contribute in part to the proinflammatory response. In LPS-stimulated BV-6 microglial cells, R-BHB was shown to decrease NF-*κ*B activation and the expression of TNF-*α*, IL-1*β*, and IL-6 [211]. When infused into the rat brain prefrontal cortex for 21 days in a model of depression (chronic unpredictable stress paradigm), R-BHB was shown to prevent the increase in TNF-*α* and the decrease in corticosterone brought about by the depression-inducing stress [212]. Peripheral injection of R-BHB was also able to decrease IL-1*β* and TNF-*α* levels in the hippocampus of rats in this depression model [213]. In bovine aorta endothelial cells stimulated with LPS, R-BHB was shown to decrease the expression of TNF-*α* and interferon [214]. The different results in different cell types and conditions clearly indicate that more research needs to be done to understand the complex regulation of cytokine production by R-BHB.

## 8. Molecular Mechanisms through Which R-BHB Restores Redox Balance

### 8.1. Activation of FOXO Transcriptional Regulators and Sirtuin Deacetylases

A major mechanism through which R-BHB restores metabolism and redox balance is through epigenetic regulation of gene expression by increasing histone beta-hydroxybutyrylation (Figure 9(c)) and inhibiting class I and IIa histone deacetylases (HDACs) to increase histone acetylation. Transcription factors and coactivators such as FOXO1 [215, 216], FOXO3a [151], and PGC-1*α* [217] are induced. Complexly, HDAC inhibitors can also lead to increased acetylation of FOXO1, which reduces its activity at the promoters of genes for gluconeogenic enzymes in the liver to decrease blood glucose levels [218]. This is likely beneficial for inhibiting the cytokine storm as discussed above. These transcriptional regulators increase mitochondrial ETC gene expression to help restore the NAD^+^/NADH. They initiate an antioxidant gene expression program together with the induction of PPP enzymes [219, 220] to restore the NADP^+^/NADPH.

In tissues such as liver, increased R-BHB levels lead to HDAC inhibition at the FOXO1 promoter and increased gene expression decreasing proinflammatory cytokine expression [216]. AMP kinase [221] and NAD^+^-dependent SIRT1 deacetylase [222] enzymes also act on FOXO1 to increase its activity. Insulin signaling through the AKT pathway inhibits FOXO1 activity [223]. The anti-inflammatory action of FOXO1 may be due in part to its activity in lung macrophages where it binds to IRF4 and stimulates the M2 state [224]. However, in tumor-localized macrophages, FOXO1 has been shown to stimulate the proinflammatory M1 state and IL-1*β* production [225]. So, the effects of FOXO1 on macrophage function appear to be dependent upon the environmental conditions. Increasing NAD^+^ levels in the cytoplasm activates SIRT2 to deacetylate glucose-6-phosphate dehydrogenase (G6PD) to increase PPP flux and NADPH production [226], linking the ratios of the pyridine nucleotide coenzyme couples. SIRT2 also deacetylates the inflammasome, inhibiting its function [227], while SIRT3 function also inhibits inflammasome activity by decreasing mitochondrial ROS levels [148]. Increased nucleocytoplasmic NAD^+^ level also increases the activity of SIRT1, which deacetylates PGC-1*α* to stimulate mitochondrial ETC function [228] leading to increased mitochondrial NAD^+^/NADH. The increased mitochondrial NAD^+^/NADH activates mitochondrial SIRT3 to deacetylate and activate mitochondrial SOD2 [229], isocitrate dehydrogenase 2 (IDH2) [230], and the 39 kD subunit of ETC complex I [231] to decrease ROS levels and decrease the mitochondrial NADP^+^/NADPH. The decreased mitochondrial NADP^+^/NADPH maintains reduced mitochondrial GSSG/GSH to prevent the glutathionylation of ETC complex I and the oxidation of cardiolipin that decrease complex I activity and decrease the matrix space NAD^+^/NADH [232]. Other important genes induced by the FOXO1 transcriptional regulator to restore metabolism and decrease inflammation and ROS production include GPR109A, lactate dehydrogenase B (LDHB), thioredoxin 2 (TXN2), PEPCK1, and the NAD^+^ synthesis genes NAMPT and NMNAT2 [233].

### 8.2. Increased Expression of PGC-1*α* and ERR-*α*

PGC-1*α* and ERR-*α* (estrogen-related receptor-*α*), a binding partner of PGC-1*α* involved in the induction of mitochondrial ETC gene expression [234], are the transcriptional regulators that are known to induce OXCT1 gene expression in myotubes to increase the levels of its gene product SCOT (Figure 9(f)) [235]. ERR-*α*, which is widely expressed, is also required for adipose tissue thermogenesis [236] and is induced by fasting, calorie restriction, cold exposure, and exercise [237, 238]. The ketogenic diet has been shown to increase PGC-1*α* levels in muscle [239], neurons [95], and brown adipose tissue [240]. These data suggest that ERR-*α* and PGC-1*α* are likely the transcriptional regulators that induce OXCT1 and ETC gene expression in the lungs during the ketogenic diet to increase ketolysis and mitochondrial biogenesis [113]. SCOT activity has been shown to be decreased by tyrosine nitration [241] and increased by tryptophan nitration [242]. SCOT activity was also inhibited by acetylation and activated by SIRT3-mediated deacetylation [243]. High-fat diets can decrease PGC-1*α* levels in the liver, decreasing its suppression of NF-*κ*B and leading to increased cytokine production [244]. Contrary to this result, a relatively high-fat diet in the presence of ketone ester was shown to increase PGC-1*α* levels and mitochondrial function in brown adipose tissue to stimulate thermogenesis [94, 245]. Therefore, the consumption of ketone ester may reverse the effects of a high-fat diet on PGC-1*α* expression in some tissues to stimulate fatty acid oxidation and prevent the accumulation of fat in tissues that is associated with negative health outcomes.

### 8.3. Metabolic Enzymes, Redox Shuttles, and Mitochondrial Uncoupling Can Restore the NAD^+^/NADH and NADP^+^/NADPH Ratios

Once the viral- and cytokine storm-induced changes in the mitochondrial NAD^+^/NADH have been partially restored through R-BHB-mediated signaling, enzyme inhibition, and upregulation of gene expression, the NAD^+^-dependent BDH1 enzyme can more effectively catalyze the conversion of R-BHB to acetoacetate. Acetoacetate is then metabolized to acetoacetyl-CoA, which is metabolized into two molecules of acetyl-CoA. As mentioned above, this pathway of acetyl-CoA synthesis becomes especially important under conditions of viral infection because PDK4 expression is upregulated leading to PDC inhibition.

#### 8.3.1. Nicotinamide Nucleotide Transhydrogenase

Nicotinamide nucleotide transhydrogenase (NNT) is an enzyme that uses energy from the mitochondrial inner membrane proton gradient to synthesize NADPH and NAD^+^ from NADP^+^ and NADH. NNT gene expression is likely induced by FOXO3a, since there are binding sites for FOXO3a in the NNT promoter [233] and since the *C. elegans* NNT homolog *nnt-1* is induced by the *C. elegans* FOXO homolog *daf-16* [246, 247]. NNT activity is likely high during times of mitochondrial ETC dysfunction, such as during a cytokine storm, as decreased mitochondrial NAD^+^/NADH and increased mitochondrial NADP^+^/NADPH stimulate NNT function in the normal NADPH-synthesizing and NADH-hydrolyzing direction.

#### 8.3.2. The Citrate-Pyruvate Shuttle and Other Mitochondrial Shuttles Modulate Cytoplasmic and Mitochondrial Redox Status

Recent evidence suggests that when glucose levels and PPP activity are low, serine and glycine are catabolized in mitochondria, which stimulates one-carbon metabolism, to generate NADPH [248]. There are mechanisms in place to use the mitochondrial matrix space-synthesized NADPH to prevent product inhibition of enzyme function, the most important being the fueling of glutathione reductase and thioredoxin reductase to combat ROS. Alternatively, the NADPH equivalents can be shuttled to the cytoplasm using the citrate-pyruvate shuttle. This involves the catabolism of glutamine and glutamate to alpha-ketoglutarate, which can lead to IDH2 functioning in the opposite direction of its normal citric acid cycle activity to oxidize NADPH and form isocitrate in the process called reductive carboxylation [249, 250]. Isocitrate can then be further metabolized to citrate. This citrate, together with other citrate molecules, such as those derived from R-BHB metabolism, gets shuttled into the cytoplasm though the mitochondrial citrate carrier protein (CIC) as part of the citrate-pyruvate shuttle (see Figure 3). In the cytoplasm, the citrate can be converted to acetyl-CoA and oxaloacetate by ATP-citrate lyase (ACLY). The acetyl-CoA can function in histone acetylation or fatty acid synthesis, while the oxaloacetate can be converted to malate by malate dehydrogenase 1 (MDH1) to restore the cytoplasmic NAD^+^/NADH. Malate can then be converted to pyruvate by NADP^+^-dependent malic enzyme (ME1), which concurrently synthesizes NADPH [248]. In the final step, the pyruvate is shuttled back into the mitochondrial matrix space, where it is metabolized by pyruvate carboxylase to form oxaloacetate. The net result is 2ATP + NADH + NADP^+^ − >2ADP + NAD^+^ + NADPH, which contributes to the restoration of the redox state. The result on the redox state is very similar to that which occurs due to the NNT reaction, except the NAD^+^ and NADPH are formed in the cytoplasm instead of the mitochondrial matrix. Increased levels of citrate and acetyl-CoA in the cytoplasm, which would likely occur as a result of increased shuttle function, inhibit glycolysis at phosphofructokinase [251] and pyruvate kinase [252], respectively, which would further aid in the restoration of NAD^+^/NADH.

In M1-polarized macrophages, citrate-pyruvate shuttle function can provide NADPH that fuels NADPH oxidase-mediated ROS production and contributes to inflammation. These M1 macrophages upregulate the expression of *cis*-aconitate decarboxylase (IRG1/ACOD1) to convert the citric acid cycle metabolite *cis*-aconitate to itaconate, which exerts anti-inflammatory actions to restrain the M1 response, by inhibiting ETC complex II activity to decrease ROS production and by activating the Nrf2 (NFE2L2) transcriptional regulator. Therefore, inhibitors of CIC and ACLY have been shown to be anti-inflammatory compounds [253], but these inhibitors may have deleterious effects on the cytoplasmic and mitochondrial redox states in other cell types. In a related redox shuttle, the citrate-malate shuttle, the cytoplasmic malate is imported into the mitochondrial matrix and therefore no cytoplasmic NADPH is synthesized. However, this shuttle is slightly more energy efficient, using the hydrolysis of only one molecule of ATP. A third shuttle system that exports citrate from the mitochondrial matrix is the citrate-alpha-ketoglutarate shuttle. In this shuttle, cytoplasmic citrate is converted into isocitrate and then further into alpha-ketoglutarate by cytoplasmic aconitase (ACO1) and isocitrate dehydrogenase 1 (IDH1), respectively, with the latter reaction synthesizing NADPH to decrease the cytoplasmic NADP^+^/NADPH [14]. The alpha-ketoglutarate can then be transported back into the mitochondrial matrix. IDH1 expression is downregulated in M1-polarized macrophages when measured 24 hours after stimulation with LPS [254] to turn off citrate-alpha-ketoglutarate shuttle flux and stimulate citrate-pyruvate shuttle function. However, two to four hours after LPS stimulation of macrophages, the alpha-ketoglutarate-dependent histone demethylase genes KDM6B [255] and PHF2 [256] are induced to increase inflammation [257]. Therefore, the citrate-alpha-ketoglutarate shuttle proteins CIC, ACO1, and IDH1 may play a role early in the M1 polarization process to provide nucleocytoplasmic alpha-ketoglutarate for the function of these histone demethylase enzymes before the shuttle is turned off.

#### 8.3.3. Mitochondrial Uncoupling Proteins

Another important mechanism through which a ketogenic diet restores the mitochondrial NAD^+^/NADH during mitochondrial ETC dysfunction is through inducing the expression of mitochondrial uncoupling proteins. The rate of NADH oxidation at complex I is normally limited by matrix space ADP levels. The presence of uncoupling proteins removes this limitation by allowing protons to flow back into the matrix space to produce heat. This increases the rate of complex I NADH oxidation to increase the mitochondrial NAD^+^/NADH. The cytoplasmic NAD^+^/NADH can also be altered by mitochondrial redox changes through malate-aspartate shuttle function. Another benefit of partial mitochondrial uncoupling is the slight decrease in the mitochondrial membrane potential that greatly decreases the generation of superoxide at ETC complexes I and III [258, 259]. One drawback of the increased expression of uncoupling proteins is the decrease in ATP generation. The ketogenic diet increases UCP1 expression in brown adipose tissue [240] and UCP2 [95], UCP4, and UCP5 [260] in the brain. Increases in uncoupling protein levels have been shown to parallel increases in PGC-1*α* levels. Administration of ketone ester to mice has been shown to increase the cytoplasmic NAD^+^/NADH and decrease the cytoplasmic NADP^+^/NADPH [16] and likely utilizes mitochondrial uncoupling and the other mechanisms listed above to restore redox balance.

### 8.4. Nrf2 Is Activated by DJ-1 during the Appropriate Redox Conditions to Restore the Redox State

The mildly elevated ROS production from R-BHB metabolism from a ketogenic diet leads to the activation of the Nrf2 transcriptional regulator [261], which stimulates antioxidant response element (ARE) gene expression. Paraquat, a redox cycling agent, which greatly increases superoxide production from ETC complex I, was shown to decrease Nrf2 levels, by a mechanism described in detail below, which was restored by the administration of R-BHB [262]. Activation of Nrf2 blunts the cytokine storm through inducing the expression of antioxidant system enzymes including heme oxygenase-1, SOD2 [263], NADP(H) quinone oxidoreductase (NQO1), gamma-glutamylcysteine synthetase (GCLC), thioredoxin (TXN), thioredoxin reductase 1 (TXNRD1) [264], and multiple enzymes for NADPH synthesis including IDH1, malic enzyme 1 (ME1), and four enzymes of the PPP including G6PD, 6-phosphogluconate dehydrogenase (PGD), transaldolase, and transketolase [265].

Nrf2 can be activated by hydrogen peroxide when the ratio of NADP^+^/NADPH is not too high or too low as shown in Figure 9(e). For Nrf2 activation to occur, superoxide produced by the ETC in the mitochondrial matrix is converted into hydrogen peroxide by SOD2. Hydrogen peroxide is then transported out of the mitochondrial matrix through aquaporins present in the inner mitochondrial membrane. In the cytoplasm, the redox-sensitive chaperone DJ-1 is activated when cysteine 106 sulfhydryl is oxidized to sulfenic acid by hydrogen peroxide. This activated form of DJ-1 is able to release KEAP1 from Nrf2 allowing Nrf2 to enter the nucleus and induce gene expression. When the NADP^+^/NADPH is too low, it prevents DJ-1 from being oxidized and activated. When the NADP^+^/NADPH is too high, cysteine 106 in DJ-1 becomes overoxidized to sulfonic acid and DJ-1 is destabilized, ubiquitinated, and degraded by the proteasome, so Nrf2 is not activated [266]. Therefore, R-BHB metabolism likely preserves the function of Nrf2 by providing the proper NADP^+^/NADPH ratio, which maintains low to moderate levels of cytoplasmic hydrogen peroxide.

### 8.5. HIF1-*α* Stabilization by RNS Leads to Proinflammatory Cytokine Production

Hypoxia-inducible factor-1*α* (HIF-1*α*) is the master transcriptional regulator of hypoxic gene expression. During normoxia, HIF-1*α* is hydroxylated on prolines, which stimulates its binding to von Hippel-Lindau (VHL) protein that targets it for proteasomal degradation. HIF-1*α* is also hydroxylated on asparagine residues by FIH-1 (factor inhibiting HIF-1-1) to prevent HIF-1*α* from binding to its coactivator CBP/p300. During hypoxia, the oxygen-dependent prolyl and asparaginyl hydroxylases are inactive stabilizing the transcriptionally active form of HIF-1*α*. HIF-1*α* can also be activated by ROS or RNS. HIF-1*α* induces the expression of glucose transporters, glycolytic enzymes, and PDK1 to inhibit PDC and shunt glycolysis-derived carbon flux away from mitochondria when oxidative phosphorylation is compromised.  Figure 9(d) shows the S-nitrosylation and activation of HIF-1*α* by peroxynitrite. S-nitrosylation of HIF-1*α* prevents the interaction with VHL for stabilization and inhibits asparagine hydroxylation for activation [267].

HIF-1*α* can also induce expression of the proinflammatory cytokines TNF-*α* and IL-6 by upregulating NF-*κ*B [268]. HIF-1*α* has been shown to be stabilized by increased pyruvate levels [269], such as those that occur following PDC inhibition, or by increased succinate levels [270]. The circadian transcriptional regulator Bmal1, which can be induced by the hormone melatonin [271], decreases HIF-1*α* stability and levels to increase mitochondrial oxidative metabolism [272]. In lung AECs, stabilization of HIF1-*α* was shown to cause ER stress and CHOP-mediated apoptosis [273]. IAV infection was shown to induce the nuclear translocation of HIF-1*α* by activating the c-Jun N-terminal kinase (JNK) signaling pathway to increase proinflammatory cytokine expression [274, 275]. However, HIF-1*α* may also play a role in suppressing IAV infection as HIF-1*α* deficiency stimulated IAV replication in AEC II cells by increasing autophagy [276]. RSV infection was shown to stabilize HIF-1*α* by increasing nitric oxide and peroxynitrite levels [277]. This resulted in increased NF-*κ*B, proinflammatory cytokine levels, and viral replication [278, 279]. Therefore, increasing levels of R-BHB to decrease RNS levels will likely be able to decrease the activation of HIF-1*α* and its downstream inflammatory mediators to mitigate SARS-CoV-2 and other respiratory viruses.

## 9. The Effects of R-BHB on Cells of the Immune System

### 9.1. Increased NADPH Can Increase or Decrease ROS Levels Depending upon the Cell Type

NADPH has roles both in the synthesis of superoxide through its role as a cofactor for NOX2 and in peroxide detoxification through its roles as cofactors for glutathione reductase and thioredoxin reductase. In most cell types, the lower K*_m_* of NADPH for the reductase antioxidant enzymes allows the antioxidant effect to predominate [280–282]. However, in some cell types such as macrophages and neutrophils, the high expression level of NOX enzymes allows ROS production to predominate. Lung AECs also have relatively high levels of NOX2, NOX4, DUOX1, and DUOX2 [9]. Future studies should address how altered NADP^+^/NADPH regulates ROS production in these cells and if increased ketone body levels increase NADPH levels in macrophages, neutrophils, and lung epithelial cells to increase NADPH oxidase activity to stimulate host defenses against pathogens.

In lung epithelial cells, knockdown of G6PD to decrease NADPH synthesis reduced NOX2 activity to decrease the antiviral response [283]. In mice, it has been shown that decreasing NADPH synthesis by inhibiting G6PD and PPP flux with 6-aminonicotinamide decreased LPS-induced inflammation in a model of acute lung injury [284]. This data is consistent with the protective effects of the NOX2 inhibitor for IAV infection [68]. The expression of SOD2 and catalase is induced by R-BHB-mediated HDAC inhibition [151], so when R-BHB increases NADPH levels to stimulate NADPH oxidase activity, it also increases ROS detoxification enzymes to prevent excessive oxidative stress that may lead to a cytokine storm. Increased NADPH levels also have been shown to stimulate antiviral immunity by decreasing the level of the NADPH sensor protein HSCARG, which is a negative regulator of NF-*κ*B transcription. Through this mechanism, increased NADPH levels were shown to increase expression of the MX1 and TNF-*α* genes to decrease human coronavirus infection [285]. One other potential proinflammatory action of the high NADPH levels from R-BHB metabolism is the reduction of dihydrobiopterin (BH2) to tetrahydrobiopterin (BH4). BH4 is an essential cofactor for nitric oxide synthases and aromatic amino acid hydroxylases [286]. So, increased NADPH levels may increase nitric oxide synthase activity leading to RNS production and inflammation. However, when BH4 levels are low, nitric oxide synthases synthesize superoxide instead of nitric oxide [287]. In this way, increased NADPH decreases ROS production as it increases RNS production. This function could potentially decrease toxic peroxynitrite levels when superoxide is limiting for its synthesis.

### 9.2. The Effects of R-BHB and Acetoacetate on Macrophage Function

Lung-resident macrophage polarization to either a proinflammatory M1 state or an anti-inflammatory M2 state is largely controlled by the cytokines secreted by the other cells present in the environment [288]. R-BHB-mediated HDAC inhibition, GPR109A signaling, and inflammasome inhibition in these cells likely increase the amounts of anti-inflammatory cytokines, such as IL-10, produced to stimulate more macrophages to the M2 state. These cytokines also influence the catabolic pathways that are activated to fuel cellular energy needs. M1 macrophages have increased glycolytic activity and lactate production due to the presence of a dysfunctional citric acid cycle [289]. Increased nitric oxide levels may inactivate citric acid cycle enzyme aconitase (ACO2) and the PDC E3 subunit dihydrolipoyl dehydrogenase (DLD). Nitric oxide also may decrease the activities of ETC complexes I, II, and IV in M1 macrophages [290]. The dysfunctional citric acid cycle resulted in the accumulation of citrate that increased fatty acid synthesis [291] and succinate that increased mitochondrial ROS production [270]. This metabolic programming in M1 macrophages likely evolved to increase proinflammatory cytokine production. M2 macrophages are programmed to oxidize pyruvate and fatty acids into acetyl-CoA for normal citric acid cycle function and oxidative phosphorylation [292], which has been shown to facilitate anti-inflammatory IL-10 secretion [293]. Since macrophages lack the enzyme BDH1, they cannot oxidize R-BHB [294], but a ketogenic diet also increases the systemic levels of the ketone body acetoacetate that can be oxidized by macrophages. The R-BHB/acetoacetate ratio produced by the liver is proportional to the mitochondrial NAD^+^/NADH ratio [295], which is normally around five [14], but varies from three to seven. In this regard, acetoacetate but not R-BHB was shown to be metabolized by macrophages to ameliorate liver fibrosis in mice [294]. Neutrophils, another type of phagocyte contributing to the cytokine storm, have few mitochondria and very low expression of ketolytic genes and therefore likely cannot catabolize R-BHB or acetoacetate to a significant extent.

### 9.3. A Ketogenic Diet Reduces Lung Inflammation by Reducing Glucose Uptake into ILC2s

A recent study examined the effects of reducing glucose levels, using a ketogenic diet, on allergen-induced lung inflammation in mice. Results showed that ILC2s in the lungs must increase their uptake of both fatty acids and glucose from the environment to elicit allergen-dependent inflammation. A ketogenic diet reduced systemic glucose levels to decrease lung ILC2 glucose uptake to prevent airway inflammation in response to the allergen [296]. The *γδ* T cell-ILC2 response activated by the ketogenic diet in mice [113] was shown to be active in human infants and children on a normal diet, where it protected them from influenza infection [297]. However, this response appears to be suppressed starting in adolescence and replaced by the ILC1 system, until it is likely reawakened by the ketogenic diet. We speculate that the higher activity of the *γδ* T cell-ILC2 response in children may be one factor responsible for the less severe symptoms when the SARS-CoV-2 virus infects individuals in this age group. The increased mortality rate of infected older adults may also be in part due to the increase in inflammation and decline in mitochondrial function and cellular NAD^+^ and NADPH levels with aging that decreases metabolic flexibility and the ability to “weather” the cytokine storm.

### 9.4. HDAC Inhibition and GPR109A Signaling Have Anti-Inflammatory Effects on Dendritic Cells

Dendritic cells play an important function in presenting antigens to T lymphocytes. Dendritic cells have a high level of oxidative metabolism until they are activated through their TLRs, at which point they switch to a primarily glycolytic metabolism [298], which is essential for their activation by providing cytoplasmic ATP for phospholipid synthesis and signals for the remodeling and expansion of the secretory system for its enhanced function in the activated state [299]. The increased rate of glycolysis in dendritic cells was also required for their secretion of interferon-*α* to mitigate IAV infection. Vaccination against IAV was able to increase glycolytic function in the dendritic cells [300]. Human monocyte-derived dendritic cells cultured with butyrate showed decreased proinflammatory cytokine and chemokine production [301]. This is likely due to the ability of HDAC inhibition and GPR109A signaling in dendritic cells to promote Treg cells [302]. The mechanism may be at least partially metabolic in nature. Dendritic cells were activated by exposure to LPS in the presence or absence of butyrate. Butyrate decreased the oxygen consumption rate and blocked the increase in extracellular acidification rate (due to lactate export) used as an indicator of the glycolytic rate [302]. Butyrate functioning as an HDAC inhibitor also inhibited the formation of dendritic cells from bone marrow stem cells [303]. Administration of ethyl pyruvate, a cell-permeable pyruvate precursor with known anti-inflammatory properties [304], was shown to inhibit the activation of dendritic cells by single-strand RNA that binds to TLR7. Ethyl pyruvate decreased glycolytic and oxidative metabolism and blocked dendritic cell activation through decreasing ERK and AKT signaling and decreasing the production of nitric oxide [298]. Therefore, ethyl pyruvate administration may be a potential therapy to block the cytokine storm during the later stages of SARS-CoV-2 infection.

### 9.5. Metabolism of R-BHB by B and T Lymphocytes May Restore Redox and Energy Levels

Increasing R-BHB levels may also stimulate the immune system by enhancing B or T lymphocyte function. Recent evidence links the ketogenic diet in mice with increasing levels of *δγ* T cells in adipose tissue [114], where the *δγ* T cells stimulate a thermogenic program [305], in part responsible for the weight loss effects frequently provided by the ketogenic diet. The ketogenic diet did not alter the level of ketone body metabolism genes in lung *γδ* T cells, but it did upregulate the expression of mitochondrial ETC genes in these cells [113]. Cells of the immune system express varied amounts of SCOT, used specifically for ketolysis, and BDH1, used for both ketogenesis and ketolysis. B and T lymphocytes possess the greatest amounts of BDH1 and SCOT of the blood cell types [134], so the ketogenic diet or exogenous ketone treatment may enhance the energy levels and redox status in these cells more effectively.

#### 9.5.1. A Ketogenic Diet Normalizes the Increased Th17/Treg Ratio That Occurs due to Disease

There are two important subtypes of CD4^+^ T lymphocytes, Th17 and Treg cells. A ketogenic diet was shown to normalize the proinflammatory Th17/Treg ratio in the blood from epileptic patients [306]. A high-fat diet may favor the expansion of Treg cells over Th17 cells as Tregs can take up and utilize fatty acids from the environment, while Th17 do not have this ability [307], so Th17 cells must synthesize fatty acids from glucose, which is present at slightly lower levels when consuming the ketogenic diet [308]. Intermittent fasting that increases R-BHB levels was also able to restore this ratio in mice with experimental autoimmune encephalomyelitis (EAE), a model of multiple sclerosis [309]. HDAC inhibition in naïve T cells leads to the expression of the FOXP3 transcriptional regulator and Treg conversion [310]. The longevity-promoting mTOR inhibitor and antiaging calorie restriction mimetic rapamycin also decreased the Th17/Treg balance. This occurred through inhibition of glycolysis in Th17 cells and stimulation of fatty acid oxidation in Tregs. Fatty acid oxidation was stimulated by AMP kinase activation [311]. A moderate dose of butyrate only induced differentiation of T cells to Treg cells when administered together with TGF-*β*1. As TGF-*β*1 level is increased by viral infection, this should not pose a problem for the treatment of SARS-CoV-2 with ketone ester. In addition, HDAC inhibition induces the expression of TGF-*β*1 in epithelial cells. However, administering a high dose of butyrate, even when added together with TGF-*β*1, was ineffective at inducing differentiation into IL-10-producing Treg cells. High doses of butyrate resulted in either normal T cells or IFN-*γ*-producing Tregs [312]. Therefore, butyrate or R-BHB may need to be present in the cell nucleus within a specific narrow concentration range for the partial inhibition of HDAC function for the optimal treatment of SARS-CoV-2.

#### 9.5.2. R-BHB Stimulates the Formation of CD8^+^ Memory T Cells

CD8^+^ memory T cells readily oxidize fatty acids and, like hepatocytes, have the rare ability to simultaneously express the genes for gluconeogenesis and ketogenesis [313]. The high level of expression of PEPCK1, the rate-limiting step in gluconeogenesis, resulted in the depletion of cellular oxaloacetate levels. Therefore, the acetyl-CoA formed from fatty acid beta-oxidation was not able to enter the Krebs cycle and was therefore used for ketogenesis. The increased R-BHB levels led to increased beta-hydroxybutyrylation of histone proteins in the FOXO1 and PGC-1*α* promoters leading to increased gene expression. Increased gluconeogenesis led to increased glucose levels that stimulated the PPP synthesis of NADPH required for the long-term protection of the CD8^+^ memory T cells against ROS [314]. Metabolic therapy with ketone ester should enhance this endogenous epigenetic program to promote the survival of CD8^+^ memory T cells to facilitate immune function when a patient is reexposed to SARS-CoV-2.

## 10. Future Perspectives

The data presented here suggest two types of clinical studies with COVID-19 patients that would provide data on the efficacy of a ketone-based metabolic therapy. These include the following:

1. A determination of forced vital capacity by spirometry of COVID-19 patients consuming ketone ester and a moderately high-fat diet (forced vital capacity is a measure of lung function based on taking a full breath and exhaling as much volume of air as possible)
2. A randomized trial of COVID-19 patients consuming ketone ester with a moderately high-fat diet with evaluations of length and severity of infection and patient mortality

A ketone-based metabolic intervention for COVID-19 patients will likely be initiated at one of the three general stages of disease progression. During all three stages, the basic treatment of raising blood ketone levels to 1 to 2 mM with exogenous ketones, increasing consumption of dietary fats, and taking enteric-coated sodium bicarbonate to buffer blood pH will likely be beneficial. The first stage is the onset of disease symptoms. During this stage, a moderate carbohydrate diet will allow normal blood glucose levels to boost immune cell function. The second stage is when the severity of symptoms requires hospitalization and/or ventilation. At this middle stage of infection, a limited carbohydrate diet would be beneficial to lower glucose metabolism and its associated proinflammatory signaling. The third stage is after ventilator use ceases and difficulty in breathing ensues. Once again, a low-carbohydrate diet at this late stage is hypothesized to facilitate beneficial anti-inflammatory processes. The expected outcomes no matter when treatment is initiated are a decrease in the incidence of progression to ARDS, protection of organs from oxidative and inflammatory damage, and increased clearance of the virus to shorten the duration of the infection. Patient data analyzed independently for the different stages of therapy initiation may yield important insights into the relative time when ketone-based metabolic intervention is most effective.

The studies suggested above, together with long-term patient monitoring, may also yield important insights into the significant post-COVID-19 patient complications. There is evidence from SARS-CoV-1 infections that psychiatric and chronic fatigue issues continued for four years after infection [315]. Unfortunately, there are anecdotes from recovered COVID-19 patients describing similar lingering morbidities. We further hypothesize that the severity of these morbidities including chronic fatigue, depression, posttraumatic stress disorders, panic disorders, and somatoform pain disorders may be blunted by a ketone-based metabolic therapy due to the mitigation of cell death and tissue damage.

## 11. Conclusions

The SARS-CoV-2 virus may become a sustained threat to global health. This review has described many of the molecular mechanisms through which an exogenous ketone-based metabolic therapy together with a moderately high-fat diet may stimulate host cell metabolism and defenses as a possible treatment to blunt the cytokine storm associated with severe SARS-CoV-2 infection. A clinical trial testing this therapy on patients with SARS-CoV-2 is warranted. In addition, further mouse IAV infection studies will aid in the determination of permissive dietary conditions under which exogenous ketone supplementation enhances immune function to facilitate viral clearance and decrease mortality.
